# Multiple genetic mutations caused by NKX6.3 depletion contribute to gastric tumorigenesis

**DOI:** 10.1038/s41598-018-35733-5

**Published:** 2018-12-04

**Authors:** Jung Hwan Yoon, Olga Kim, Jung Woo Eun, Sung Sook Choi, Hassan Ashktorab, Duane T. Smoot, Suk Woo Nam, Won Sang Park

**Affiliations:** 1 Department of Pathology, College of Medicine, The Catholic University of Korea, 222 Banpo-daero, Seocho-gu, Seoul, 06591 South Korea; 2 Functional RNomics Research Center, College of Medicine, The Catholic University of Korea, 222 Banpo-daero, Seocho-gu, Seoul, 06591 South Korea; 3 College of Pharmacy, Sahmyook University, Hwarangro 815, Nowon-gu, Seoul, 139-742 South Korea; 4 Department of Medicine, Howard University, Washington, District of Columbia 20060 USA; 5 Department of Medicine, Meharry Medical Center, Nashville, TN 37208 USA

## Abstract

NKX family members are involved in a variety of developmental processes such as cell fate determination in the central nervous system, gastrointestinal tract, and pancreas. However, whether NKX6.3 contributes to gastric carcinogenesis remains unclear. The objective of this study was to examine roles of NKX6.3 depletion in mutagenesis and gastric carcinogenesis, focusing on its effects on genetic alterations and expression of genes. Our results revealed that NKX6.3 depletion induced multiple genetic mutations in coding regions, including high frequency of point mutations such as cytosine-to-thymine and guanine-to-adenine transitions caused by aberrant expression of AICDA/APOBEC family in human gastric epithelial cells. Interestingly, NKX6.3 downregulated *AICDA/APOBEC family, NFκB*, and *CBFβ* genes by acting as a transcription factor while inhibiting deaminase activity in gastric epithelial cells. Functional relevance of NKX6.3 was validated in xenograft mice injected with NKX6.3 depleting cells. NKX6.3 depletion resulted in tumor formation and mutations of tumor-associated genes, including p53 and E-cadherin. Moreover, expression levels of NKX6.3 and its target genes were analyzed in tumors derived from mice implanted with NKX6.3 depleting cells and tissue samples of gastric cancer patients. Our results indicate that NKX6.3 depletion in gastric epithelial cells activates AICDA/APOBEC family, leading to accumulation of genetic mutations and eventually driving the development of gastric cancers.

## Introduction

Although genetic and epigenetic alterations in oncogenes and tumor suppressor genes are known to be responsible for the development and progression of gastric cancer^1^, this cancer remains the seventh most common cancer and the third leading cause of cancer-related death worldwide^2^. Recurrent mutations in gene coding regions and detailed molecular characterization of gastric cancers have been recently identified^3–5^. The majority of gastric cancers are associated with *Helicobacter pylori* infection. *H. pylori* seems to cause increased point mutation rates in mouse models^6^ and elevated expression of activation-induced cytidine deaminase (AICDA)^7^ belonging to apolipoprotein B mRNA editing catalytic polypeptide (APOBEC) family of proteins^8^.

NKX family members are involved in a variety of developmental processes such as cell fate determination in the central nervous system, gastrointestinal tract, and pancreas^9^. NKX6.3 is expressed in the epithelium of the most distal region of the stomach^9,10^. Previously, we have reported that NKX6.3 functions as a master regulator of gastric differentiation by modulating Sox2 and Cdx2 expression^11^. It also acts as a gastric tumor suppressor by inhibiting cell proliferation and inducing apoptosis^12^. In addition, loss of expression and allelic deletion of NKX6.3 have been frequently detected in gastric cancers^12^. However, whether NKX6.3 contributes to gastric carcinogenesis remains unclear. Therefore, the objective of this study was to examine roles of NKX6.3 depletion in mutagenesis and gastric carcinogenesis, focusing on its effects on genetic alterations and expression of genes.

Here, we found that NKX6.3 depletion enhances genetic mutations by regulating expression of the genes involved in mutagenesis, including the AICDA/APOBEC family, in gastric epithelial cells. This study provides new insight into molecular gastric carcinogenesis, implicating NKX6.3 depletion as a critical event.

## Results

### Genetic alterations in gastric cancers from TCGA and COSMIC database

**Figure 1:**
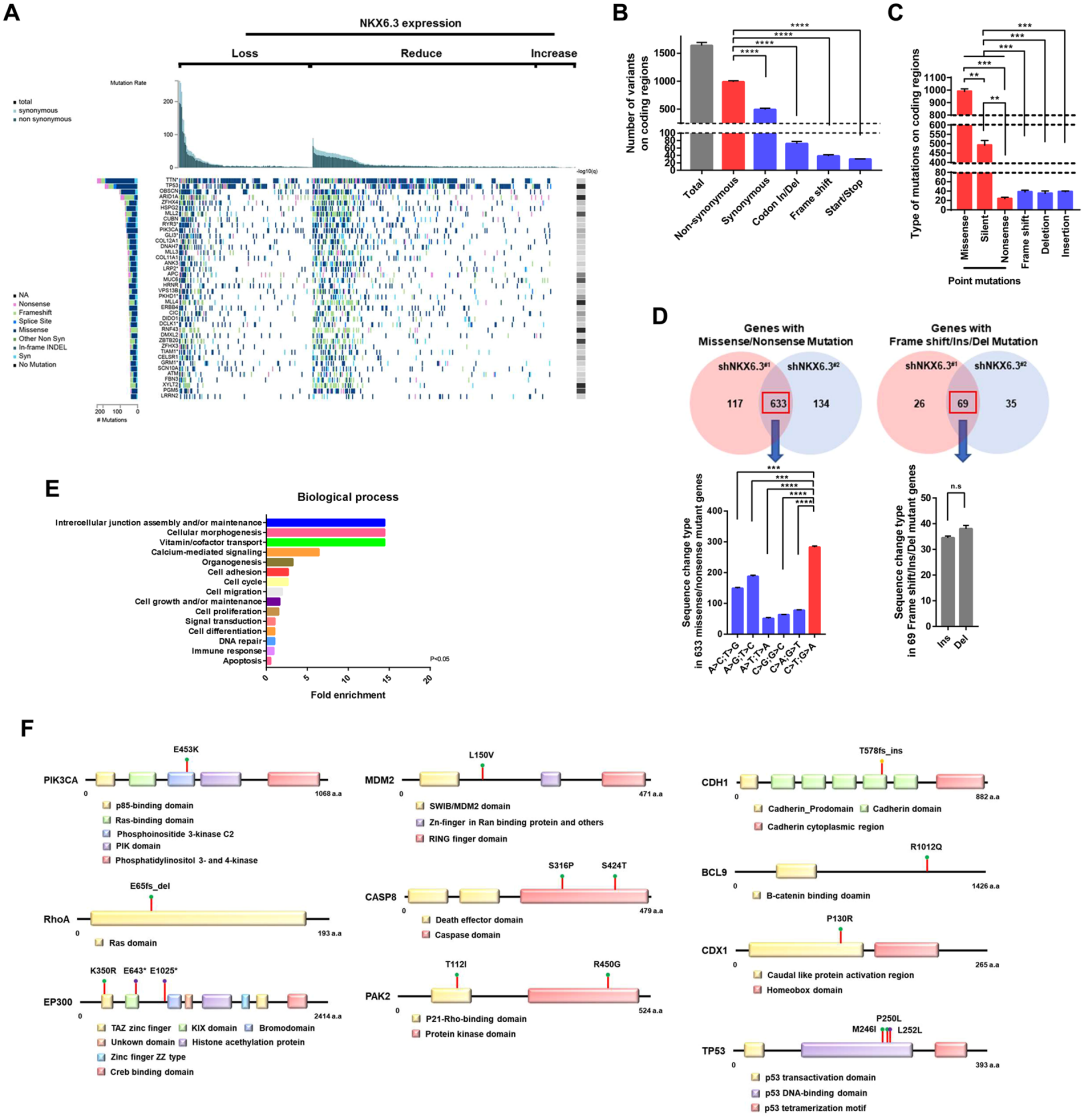
Depletion of NKX6.3 enhances mutations in coding regions. (**A**) Relationship between NKX6.3 expression and mutations in 395 gastric cancers from TCGA dataset. (**B**) Number of variants in gene coding regions in HFE-145^shNKX6.3#1^ and HFE-145^shNKX6.3#2^ cells compared to HFE-145^shCtrl^ cells. Error bars, SD. *P* values were derived from *t* tests. ****P < 0.0001. (**C**) Types of mutations observed in HFE-145^shNKX6.3#1^ and HFE-145^shNKX6.3#2^ cells. Error bars, SD. *P* values were derived from *t* tests. **P < 0.01; ***P < 0.001. **(D)** Left, Venn diagram of NKX6.3 depletion-induced mutant genes with missense/nonsense mutations, mainly G or C to A or T base pair transitions. Error bars, SD. *P* values were derived from *t* tests. ***P < 0.001; ****P < 0.0001. Right, NKX6.3 depletion-induced mutant genes with Frame shift/Ins/Del mutations in coding regions. Error bars, SD. *P* values were derived from *t* tests. n.s, not significant (*P* > 0.05). **(E)** Mutant gene signatures were involved in 15 biological processes in FunrichDB. (**F**) Mutation sites of *PIK3CA*, *RhoA*, *EP300*, *TP53*, *MDM2*, *CASP8*, *PAK2*, *CDH1*, *BCL9*, and *CDX1* induced by NKX6.3 depletion.

To determine whether defects in *NKX6.3* gene might lead to genetic alterations in gastric cancers, TCGA and COSMIC data were analyzed. In 395 gastric cancers, cases without NKX6.3 expression showed significantly higher mutation rates than those with NKX6.3 expression (Fig. 1A). Notably, most mutations in hypermutated genes were detected in gastric cancers with reduced or loss of NKX6.3 expression (Fig. 1A). Because TCGA data did not provide NKX6.3 expression level in 363 corresponding non-neoplastic gastric mucosae, we analyzed NKX6.3 expression levels and mutations rates in 32 gastric cancer samples out of 395 TCGA tumors. Compared with HFE-145 immortalized gastric epithelial cells (fold change, mean ± SD: 1.00 ± 0.02) and non-neoplastic gastric mucosae (fold change, 0.91 ± 0.18), nine cancer cell lines (fold change, 0.23 ± 0.04, *P* < 0.01) and gastric cancer tissues (fold change, 0.14 ± 0.05, *P* < 0.01) showed reduced NKX6.3 expression (Supplementary Fig. S1A). In addition, NKX6.3 expression was inversely correlated with mutation rates in 32 TCGA gastric cancer tissues (Supplementary Fig. S1B, Spearman rank correlation test; *P* < 0.0001). As shown in Supplementary Fig. S1C, a high frequency of G to A transition mutations were observed while coding region mutations in genes including *TTN, MUC16*, and *FAT4* were detected mainly in cases with reduced or loss of NKX6.3 expression (Supplementary Fig. S1D). COSMIC data from 22 gastric cancer cell lines demonstrated frequent coding region point mutations, especially in genes such as *TTN*, *MUC16*, and *TP53* (Supplementary Fig. S1E).

### Depletion of NKX6.3 leads to genetic mutations in coding regions

Next, we investigated whether depletion of NKX6.3 might induce genetic alterations in gastric epithelial cells. We performed whole genome sequencing for HFE-145^shCtrl^, HFE-145^shNKX6.3#1^, and HFE-145^shNKX6.3#2^ cells with a mean depth of 42.9x, 44.8x, and 39.9x, respectively (98.4%, 98.4%, and 98.3% of the reference human genome were covered by ≥10x, respectively, Supplementary Table S1A,B). Compared to the reference sequence of human genome (hg19), HFE-145^shCtrl^ cells showed 3,544,930 genetic variants, including polymorphism and mutations, possibly caused by serial culture and immortalization. Of these, 386 variants resulted in amino acid changes (data not shown). In HFE-145^shNKX6.3#1^ and HFE-145^shNKX6.3#2^ cells, 3,501,785 and 3,492,917 genome-wide genetic variants were observed, respectively, including 386 variants detected in HFE-145^shCtrl^ cells (Supplementary Table S1C). Additionally, 688,704 different genetic variants were found between HFE-145^shCtrl^ and HFE-145^shNKX6.3#1^ cells and 686,632 different genetic variants were found between HFE-145^shCtrl^ and HFE-145^shNKX6.3#2^ cells. Of these genetic variants, 674,571 were overlapped between HFE-145^shNKX6.3#1^ and HFE-145^shNKX6.3#2^ cells. (data not shown).

In HFE-145^shNKX6.3#1^ and HFE-145^shNKX6.3#2^ cells, 1,635.5 ± 39.5 genetic mutations were newly identified in the coding regions on whole chromosomes, including non-synonymous and synonymous, codon insertion/deletion, frame shift, and start/stop codon mutations (Supplementary Fig. S2A) compared to HFE-145^shCtrl^ cells (Fig. 1B and Supplementary Table S2). Among these mutations, a high frequency (93%) of point mutations such as missense, silent, and nonsense mutations rather than frameshift, deletion, and insertion were found. The percentage of missense mutations in point mutations was higher than that of silent and nonsense mutations (Fig. 1C). In addition, substitutions from C to T and from G to A showed high percentages (Supplementary Fig. S2B). In detail, missense and nonsense mutations were found in coding regions of 750 genes in HFE-145^shNKX6.3#1^ cells and 767 genes in HFE-145^shNKX6.3#2^ cells. Of them, 633 genes were overlapped. Mutations in these overlapped genes were found to be mainly C to T and G to A transitions (54.7%). In addition, frame shift, insertion, and deletion mutations were observed in coding regions of 95 genes in HFE-145^shNKX6.3#1^ and 104 genes in HFE-145^shNKX6.3#2^ cells. Of them, 69 genes were overlapped (Fig. 1D). Next, we analyzed the associated biological pathway using FunRich software. Results of analysis showed that NKX6.3 depletion-induced mutant genes including *TP53*, *RhoA*, *EP300*, and *PIK3CA* were involved in biological pathways such as intracellular junction assembly and/or maintenance, cellular proliferation, and apoptosis (Fig. 1E, Supplementary Fig. S2C and Supplementary Table S3). Mutation sites of these genes are shown in Fig. 1F. These results strongly indicate that depletion of NKX6.3 can lead to genetic mutations in several genes involved in cell growth, cell proliferation, and apoptosis.

### NKX6.3 downregulates *APOBEC gene family* at transcriptional level

**Figure 2:**
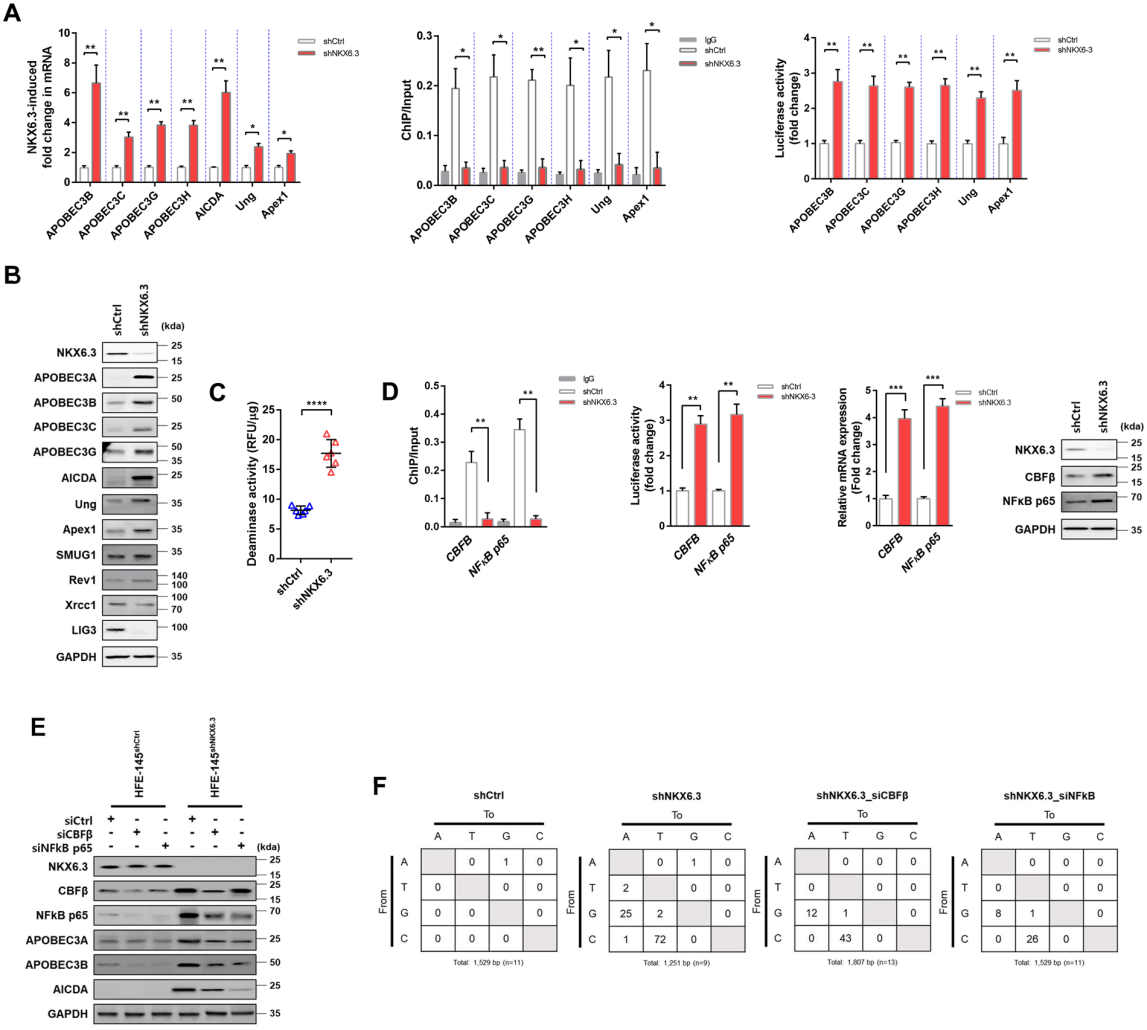
NKX6.3 regulates AICDA/APOBEC gene family expression. (**A**) Real-time RT-PCR (left), ChIP-qPCR (middle), and luciferase activity (right) analysis of *APOBEC family*, *AICDA*, *Ung*, and *Apex1* in NKX6.3 depleted cells (n = 3). Error bars, SD. *P* values were derived from *t* tests. *P < 0.05, **P < 0.01. (**B**) Immunoblot analysis of APOBEC3A, 3B, 3C, 3G, AICDA, and Ung protein expression in NKX6.3 depleted cells. (**C**) Deaminase activity in HFE-145^shNKX6.3^ cells by FRET assay (n = 3). Error bars, SD. *P* values were derived from *t* tests. ****P < 0.0001. (**D**) ChIP-qPCR (upper left), luciferase activity (upper right), real-time RT-PCR (lower left), and immunoblot (lower right) analyses of *CBFβ* and *NFκB p65* genes in NKX6.3 depleted HFE-145^shNKX6.3^ cells (n = 3). Error bars, SD. *P* values were derived from *t* tests. **P < 0.01; ***P < 0.001. (**E**) Protein expression of AICDA/APOBEC gene family in HFE-145^shNKX6.3^ cells with *CBFβ* or *NFκB p65* knockdown. (**F**) In 3D-PCR, somatic mutation in *TP53* coding region (n = 11 for shCtrl, n = 9 for shNKX6.3, n = 13 for shNKX6.3 with siCBFβ, n = 11 for shNKX6.3 with siNFκB).

To examine whether NKX6.3 might be involved in regulating AICDA/APOBEC gene family expression, we analyzed mRNA expression of *AICDA/APOBEC gene family*, *AICDA*, *Ung*, and *Apex1* genes in AGS, MKN1, and HFE-145 cells. NKX6.3 depletion in HFE-145^shNKX6.3^ dramatically increased expression levels of *APOBEC gene family*, *AICDA*, *Ung*, and *Apex1* genes whereas stable NKX6.3 expression in AGS^NKX6.3^ and MKN1^NKX6.3^ cells significantly reduced mRNA expression levels of these genes (Fig. 2A and Supplementary Fig. S3A). To determine binding of NKX6.3 to promoter upstream sequences of *APOBEC gene family* in HFE-145^shCtrl^, HFE-145^shNKX6.3^, AGS^Mock^, MKN1^Mock^, AGS^NKX6.3^, and MKN1^NKX6.3^ cells, chromatin immunoprecipitation (ChIP) assay was performed followed by qPCR. Interestingly, there were 3, 7, 4, 3, 6, and 4 putative NKX6.3 binding motifs (TAAT) in promoter regions from −0.5 kb to −3.5 kb relative to the transcription start site of *APOBEC3B, 3C, 3G, 3H, Ung*, and *Apex1* genes, respectively (Supplementary Fig. S3C). NKX6.3 binding activity to these *APOBEC family* genes in HFE-145^shCtrl^, AGS^NKX6.3^, and MKN1^NKX6.3^ cells was clearly observed (Fig. 2A and Supplementary Fig. S3D). Results of luciferase activity assay showed that mRNA expression levels of these genes were significantly increased in HFE-145^shNKX6.3^ cells but significantly decreased in AGS^NKX6.3^ and MKN1^NKX6.3^ cells (Fig. 2A and Supplementary Fig. S3E). In addition, NKX6.3 depletion markedly induced expression of APOBEC3A, 3B, 3C, 3G, AICDA, and Ung proteins whereas ectopic expression of NKX6.3 reduced their expression levels (Fig. 2B and Supplementary Fig. S3B).

To probe the biological significance of NKX6.3 as a transcriptional regulator of APOBEC gene family, effects of NKX6.3 on deaminase activity in HFE-145, AGS, and MKN1 cells were determined using fluorescence resonance energy transfer (FRET)-based assay. Results showed that deaminase activities in HFE-145^shNKX6.3^, AGS^Mock^, and MKN1^Mock^ cells that expressed AICDA/APOBEC gene family were increased. However, stable NKX6.3 expression in these cell lines resulted in significant decreases in deaminase activity (Fig. 2C and Supplementary Fig. S3F). Thus, we can conclude that NKX6.3 transcriptionally downregulates expression of *AICDA/APOBEC gene family*, *Ung*, and *Apex1* in gastric epithelial cells.

### NKX6.3 regulates *CBFβ* and *NFκB* at transcriptional level

Core-binding factor subunit beta (CBFβ) is required for *APOBEC3* gene expression^13^ while NFκB is required for the *AICDA* gene expression^7^. Since a high frequency of mutations were found in gastric cancer tissues and cell lines with loss of NKX6.3 expression, we hypothesized that NKX6.3 could modulate the expression of *CBFβ* and *NFκB*. As expected, expression levels of *CBFβ* and *NFκB* were inversely associated with levels of NKX6.3 expression but positively associated with mutation rates in TCGA datasets (Supplementary Fig. S4A). Importantly, NKX6.3 binding activities to promoter regions of *CBFβ* and *NFκB p65* genes were observed in HFE-145^shCtrl^, AGS^NKX6.3^, and MKN1^NKX6.3^ cells (Fig. 2D and Supplementary Fig. S4B). Both mRNA and protein expression levels of CBFβ and NFκB p65 were significantly increased in HFE-145^shNKX6.3^ cells but decreased in NKX6.3-expressing cells based on luciferase activity assay, real-time RT-PCR, and immunoblot analysis (Fig. 2D and Supplementary Fig. S4C–F). In addition, knockdown of *CBFβ* or *NFκB p65* significantly decreased mRNA and protein expression of AICDA/APOBEC gene family and deaminase activity in HFE-145^shNKX6.3^ cells (Fig. 2E and Supplementary Fig. S5A,B). Next, we determined 3D-PCR error rate using the first PCR amplification corresponding to 139 bp to confirm the effect of *CBFβ* or *NFκB* p65 on NKX6.3 depletion-induced somatic mutations in *TP53* coding region in HFE-145^shNKX6.3^ cells. By decreasing the denaturation temperature (Td), it was found that 87.9 °C was the minimal temperature allowing amplification of *TP53* in HFE-145^shCtrl^ cells. By contrast, 3D-PCR could amplify *TP53* gene in HFE-145^shNKX6.3^ cells at temperatures as low as 85.6 °C. As expected, knockdown of *CBFβ* or *NFκB p65* in HFE-145^shNKX6.3^ was able to recover DNA at 86.6 °C, which was a higher temperature than that of NKX6.3-depleted cells (Supplementary Fig. S5C). In addition, 3D-PCR products from amplification at Td of 87.9, 85.6, and 86.6 °C were cloned and up to 11, 9, 13, and 11 clones were sequenced per sample. Knockdown of *CBFβ* or *NFκB p65* reduced NKX6.3 depletion-induced somatic mutations in *TP53* coding region of HFE-145^shNKX6.3^ cells (Fig. 2F and Supplementary Fig. S5D). Thus, NKX6.3 might be able to protect gastric epithelial cells against AICDA/APOBEC gene family-induced somatic mutations by downregulating CBFβ and NFκB expression.

### NKX6.3 depletion induces AICDA/APOBEC mutagenesis pattern

**Figure 3:**
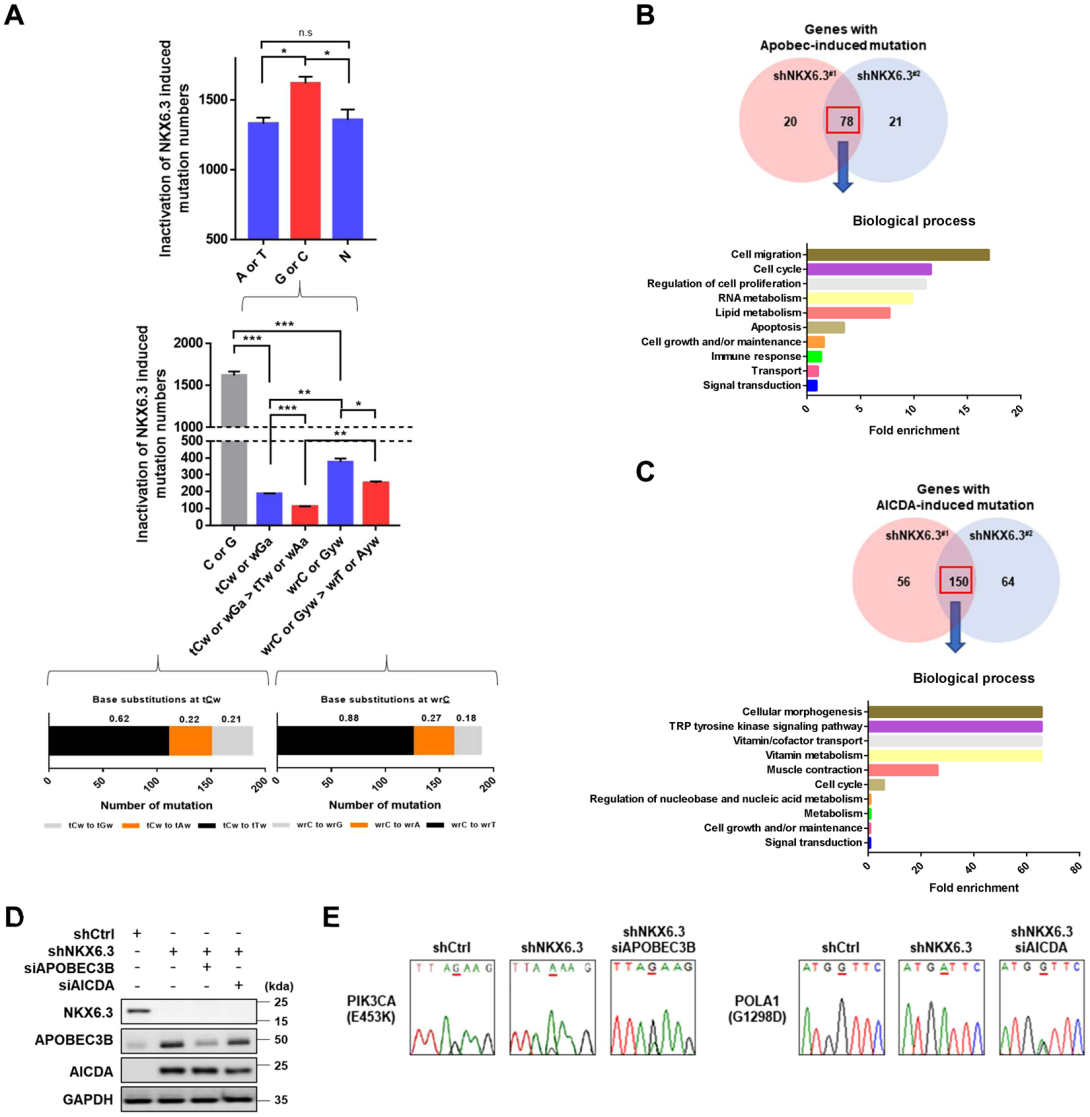
NKX6.3 depletion induces AICDA/APOBEC mutagenesis pattern. (**A**) An APOBEC mutation pattern with the highest enrichment of C to T and C to G changes on the tCw motif (low left), a strong AICDA mutation pattern with the highest enrichment on the wrC motif, and a strong preference for C to T and C to A changes on the wrC motif in NKX6.3 depleted cells (low right). Error bars, SD. *P* values were derived from *t* tests. n.s, not significant (*P* > 0.05). *P < 0.05; **P < 0.01; ***P < 0.001. (**B**) Venn diagram of APOBEC-induced mutant genes in NKX6.3 depleted cells (upper) and gene set enrichment of APOBEC-induced mutant genes (lower). (**C**) Venn diagram of AICDA-induced mutant genes in NKX6.3 depleted cells (upper) and gene set enrichment of AICDA-induced mutant genes (lower). (**D**,**E**) Knockdown of APOBEC3B and AICDA in HFE-145 cells with transfected *shNKX6.3* (D), knockdown of APOBEC3B and AICDA partially recovered NKX6.3 depletion-induced mutation in *PIK3CA* (E, left) and *POLA1* genes (E, right).

Because NKX6.3 depletion could stimulate the entire spectrum of mutations (Fig. 1), we next evaluated AICDA/APOBEC mutation pattern in HFE-145^shNKX6.3^ cells. The strength of AICDA/APOBEC mutation patterns in C and G from genome-wide analysis of coding regions in HFE-145^shNKX6.3^ cells is shown in Fig. 3A. C to T and C to G changes in tCw motif of AICDA/APOBEC mutation in HFE-145^shNKX6.3^ cells showed the highest enrichment (Fig. 3A and Supplementary Table S4). In addition, a strong AICDA mutation pattern with the highest enrichment in wrC motif and a strong preference for C to T and C to A changes in wrC motif of AICDA/APOBEC mutation in NKX6.3-depleted cells were found (Fig. 3A and Supplementary Table S4). Furthermore, APOBEC- and AICDA-induced mutations were mainly detected in coding regions and 3′-UTRs (Supplementary Fig. S6A,B). Of mutations associated with NKX6.3 depletion, APOBEC and AICDA-induced mutations were found in 78 and 150 genes, respectively (Fig. 3B,C). According to related biological pathways, we analyzed gene set enrichment of APOBEC- and AICDA-induced mutant genes. Frequent mutations in genes involved in Nef and signal transduction, adrenoceptors and VEGFR3 signaling in lymphatic endothelium (including *TP53*, *PIK3CA*, *ELMO1*, and *SMAD2*), and genes involved in proline catabolism, DNA replication initiation, and NAD phosphorylation and dephosphorylation (including *YWHAB*, *POLA1*, *PRIM2*, and *FFAR1*) were found (Fig. 3B,C, Supplementary Fig. S6C,D, and Supplementary Table S5). Knockdown of APOBEC3B and AICDA partially recovered NKX6.3 depletion-induced mutations of *PIK3CA* and *POLA1* genes in HFE-145^shNKX6.3^ cells (Fig. 3D,E). These results suggest that NKX6.3 depletion can lead to a variety of mutations, including APOBEC- and AICDA-mutation patterns.

### Effects of NKX6.3 depletion on apoptosis and invasion

**Figure 4:**
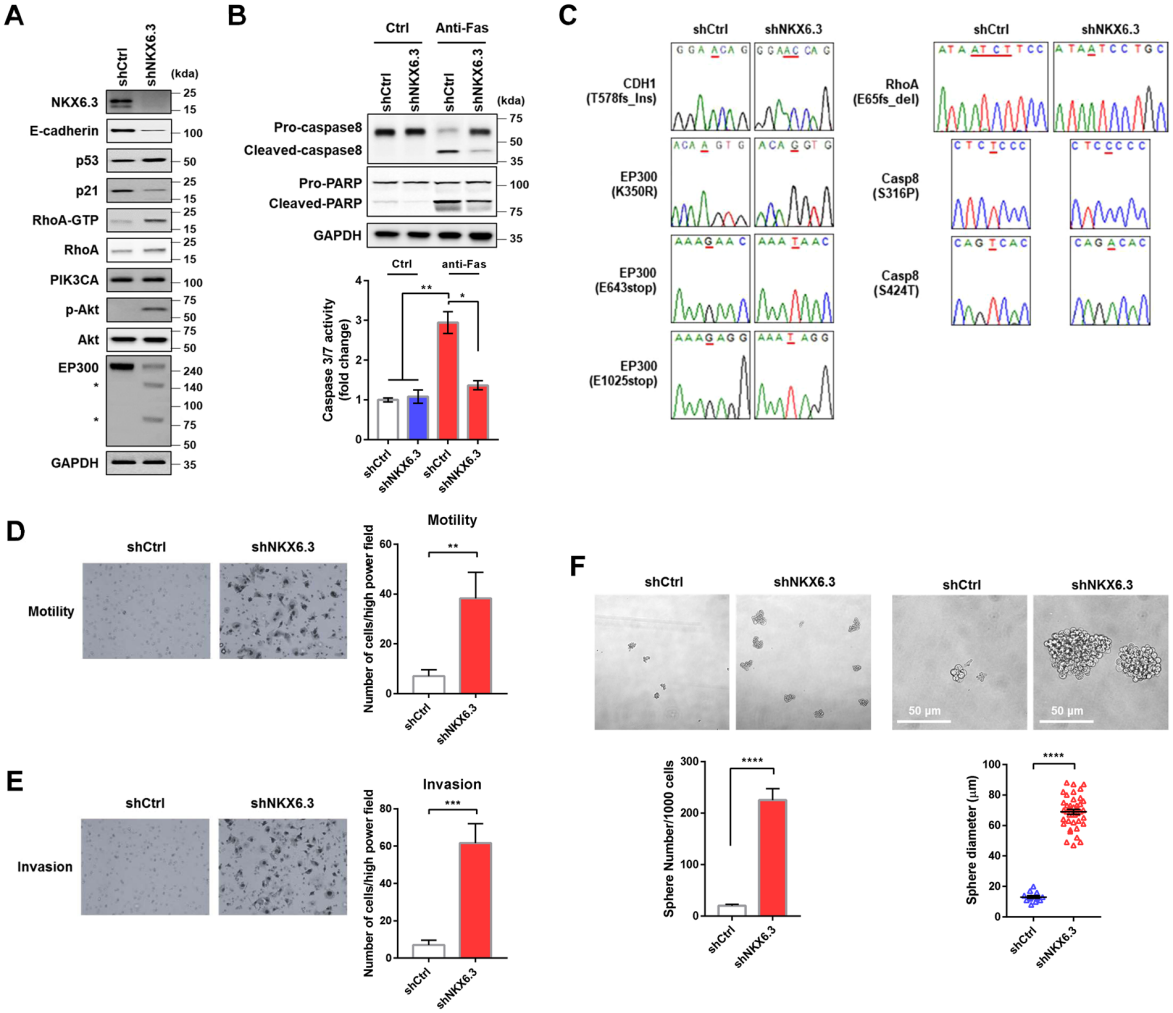
Effects of NKX6.3 depletion on apoptosis and invasion. (**A**) Immunoblot analysis of NKX6.3 depletion-induced mutant gene expressions in HFE-145^shCtrl^ and HFE-145^shNKX6.3^ cells. (**B**) Caspase 3/7 activity in HFE-145^shCtrl^ and HFE-145^shNKX6.3^ cells were treated with anti-Fas (n = 3). Error bars, SD. *P* values were derived from *t* tests. *P < 0.05; **P < 0.01. **(C)** Mutations of *CDH1*, *RhoA*, *EP300*, and *CASP8* gene in NKX6.3 depleted cells. **(D)** Cell migration activity in NKX6.3 depleted cells by transwell chemotaxis assays (n = 3). Error bars, SD. *P* values were derived from *t* tests. **P < 0.01. **(E)** Invasiveness of NKX6.3 depleted cells by Matrigel invasion assay (n = 3). Error bars, SD. *P* values were derived from *t* tests. ***P < 0.001. **(F)** Sphere number (left, n = 3) and size (right, n = 13, 40 spheres per group) in HFE-145^shCtrl^ and HFE-145^shNKX6.3^ cells based on sphere forming assay. Error bars, SD. *P* values were derived from *t* tests. ****P < 0.0001.

Since NKX6.3 was associated with mutations in *CDH1*, *TP53*, *RhoA*, *PIK3CA*, and *EP300* genes (Fig. 1), we examined the expression of mutant genes and effects of NKX6.3 depletion on apoptosis and invasion. When we examined the expression and activity of E-cadherin, TP53, RhoA, PIK3CA, and EP300 in HFE-145^shNKX6.3^ cells, NKX6.3 depletion reduced E-cadherin and p21 expression, increased RhoA expression and Akt phosphorylation, and induced decreased and fragmented expression of EP300 possibly due to *p53*, *RhoA*, or *PIK3CA* mutations (Fig. 4A). In addition, increased expression of cleaved PARP and increased caspase-3/7 activity after anti-Fas treatment were significantly inhibited in NKX6.3-depleted cells possibly due to *caspase-8* mutation (Fig. 4B). Furthermore, mutations of these genes in HFE-145^shNKX6.3^ cells were confirmed by sequencing (Fig. 4C).

Next, to determine the effect of NKX6.3 depletion on cell migration and invasion, we performed transwell chemotaxis and Matrigel invasion assays. Cell migration and invasion activities were significantly increased in HFE-145^shNKX6.3^ cells (Fig. 4D,E). In tumor sphere assay *in vitro*, depletion of NKX6.3 dramatically increased sphere number and size in HFE-145 cells (Fig. 4F).

**Figure 5:**
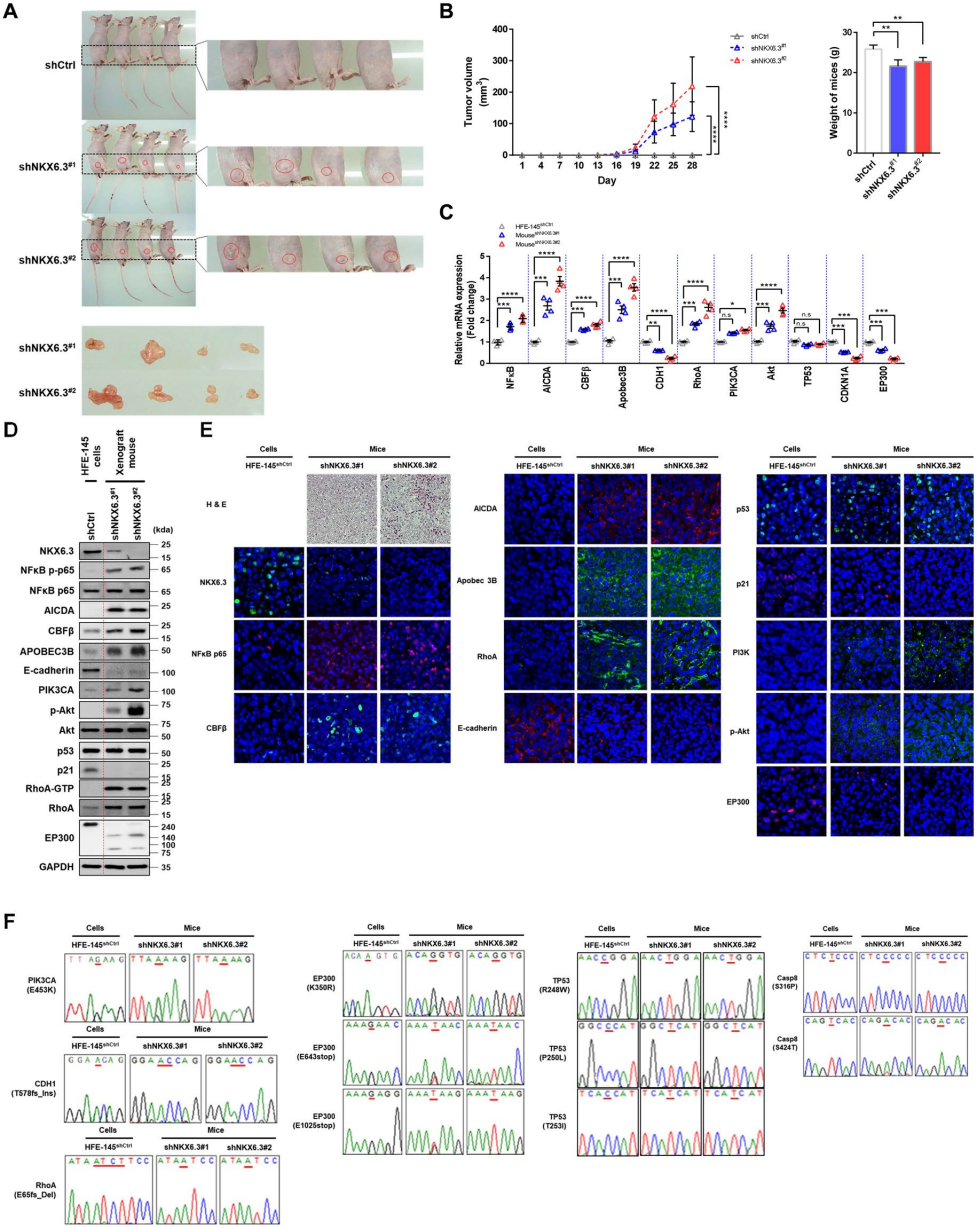
Depletion of NKX6.3 drives gastric tumorigenesis in xenograft model. (**A**) All mice implanted with HFE-145^shNKX6.3#1^ and HFE-145^shNKX6.3#2^ cells developed tumors whereas all mice subcutaneously injected with HFE-145^shCtrl^ cells did not (each group: n = 4). (**B**) Tumor volumes and weight in mice implanted with HFE-145^shNKX6.3#1^ and HFE-145^shNKX6.3#2^ cells (each group: n = 4). Error bars, SD. *P* values were derived from *t* tests. **P < 0.01; ****P < 0.001. (**C,D**) mRNA (C) and protein (D) expression levels of CDH1, CDKN1A, EP300, NFκB p65, AICDA, CBFβ, APOBEC3B, PIK3CA, p-Akt, and RhoA in HFE-145^shNKX6.3#1^- and HFE-145^shNKX6.3#2^-induced xenograft tumors (n = 4). Error bars, SD. *P* values were derived from *t* tests. n.s, not significant (*P* > 0.05). *P < 0.05; **P < 0.01; ***P < 0.001; ****P < 0.0001. (**E**) Immunofluorescent staining for NKX6.3, NFκB p65, CBFβ, AICDA, APOBEC3B, RhoA, E-cadherin, p53, p21, PI3K, p-Akt, and EP300 in HFE-145^shCtrl^ cells or HFE-145^shNKX6.3#1^- and HFE-145^shNKX6.3#2^-induced xenograft tumors. (**F**) Wild and mutant sequences of *PIK3CA*, *CDH1*, *RhoA*, *EP300*, *TP53*, and *CASP8* gene in HFE-145^shCtrl^ cells or HFE-145^shNKX6.3#1^- and HFE-145^shNKX6.3#2^-induced xenograft tumors.

We next investigated whether NKX6.3 regulated gastric tumorigenesis using xenograft mouse model (Fig. 5A). Briefly, 5 × 10^6^ NKX6.3 depleted cells (HFE-145^shNKX6.3#1^ and HFE-145^shNKX6.3#2^ cells) or non-target control (HFE-145^shCtrl^) were inoculated subcutaneously into the lower flank of BALB/C nude mice. Interestingly, mice implanted with HFE-145^shNKX6.3#1^ and HFE-145^shNKX6.3#2^ cells developed tumors (n = 4/4, 100%, respectively) whereas no mice subcutaneously injected with HFE-145^shCtrl^ cells developed tumors (n = 0/4, 0%) (Fig. 5A,B). Mice injected with HFE-145^shNKX6.3#1^ and HFE-145^shNKX6.3#2^ cells showed significantly reduced body weights compared to HFE-145^shCtrl^ cells injected mice (Fig. 5B). In real-time RT-PCR, immunoblot, and immunofluorescent analyses, tumors derived from mice implanted with HFE-145^shNKX6.3#1^ and HFE-145^shNKX6.3#2^ cells exhibited reduced E-cadherin and p21 expression but increased activity of NFκB p65, AICDA, CBFβ, APOBEC3B, RhoA and p-Akt with decreased and fragmented expression of EP300 (Fig. 5C–E). Sequencing HFE-145^shNKX6.3#1^ and HFE-145^shNKX6.3#2^ tumors confirmed that NKX6.3 depletion caused mutations in *RhoA*, *PIK3CA*, *TP53*, *CDH1*, *EP300* genes (Fig. 5F). These results suggest that NKX6.3 depletion may inhibit apoptosis and increase invasion and sphere-forming activity due to mutations in genes associated with apoptosis and invasion, subsequently contributing to gastric tumorigenesis.

### NKX6.3 expression is inversely correlated with AICDA/APOBEC gene family and DNA repair genes in both gastric cancers and non-cancerous gastric mucosae

To further confirm that NKX6.3 expression could regulate the expression of *AICDA, NFκB p65, CBFβ, APOBEC3A* and *APOBEC3B*, mRNA expression levels of these genes were compared with NKX6.3 expression in 55 non-neoplastic gastric mucosae. NKX6.3 expression was inversely correlated with expression of these genes (Supplementary Fig. S7A) while expression levels of *AICDA*, *APOBEC3A*, and *APOBEC3B* were positively correlated with expression of *NFκB p65* and *CBFβ* (Supplementary Fig. S7B).

**Figure 6:**
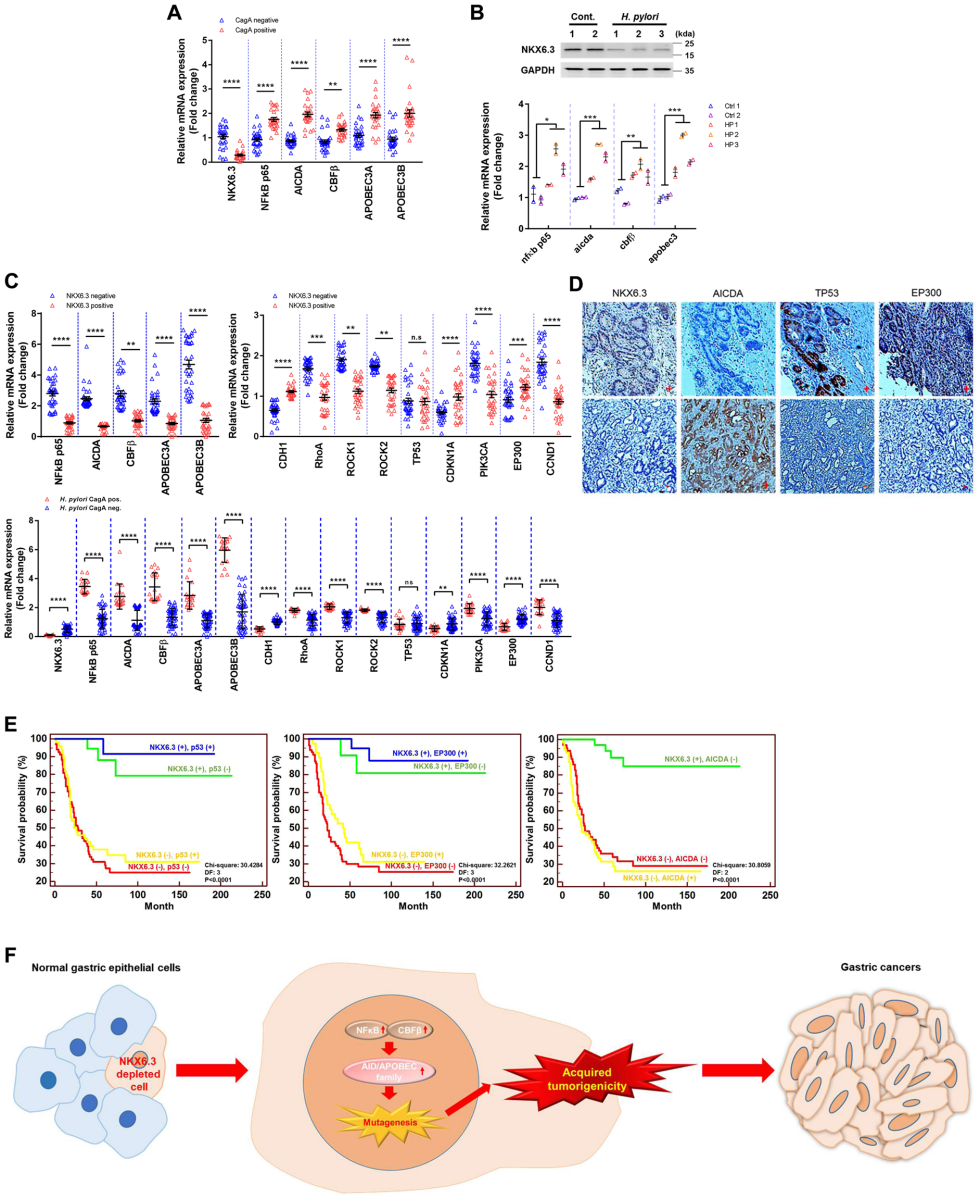
NKX6.3 expression is inversely correlated with AICDA/APOBEC gene family and DNA repair genes in both gastric cancers and non-cancerous gastric mucosae. (**A**) mRNA expression levels of NKX6.3, *AICDA*, *NFκB p65*, *CBFβ*, *APOBEC3A* and *APOBEC3B* in *H. pylori* CagA positive gastric mucosae compared to those in *H. pylori* CagA negative gastric mucosae (n = 55, triplicated experiments). Error bars, SD. *P* values were derived from *t* tests. **P < 0.01; ****P < 0.0001. (**B**) Protein levels of NKX6.3 and mRNA expression levels of *AICDA*, *NFκB p65*, *CBFβ*, and *APOBEC3* in gastric mucosa tissues from *H. pylori*-infected mice (Ctrl, n = 2; H.P, n = 3, triplicated experiments). Error bars, SD. *P* values were derived from *t* tests. *P < 0.05; **P < 0.01; ***P < 0.001. (**C**) mRNA expression levels of *AICDA*, *NFκB p65*, *CBFβ*, *APOBEC3A*, *APOBEC3B*, *CDH1*, *CDKN1A*, *EP300*, *RhoA*, *ROCK1*, *ROCK2*, *PIK3CA*, and *CCND1* in gastric cancers with negative and positive NKX6.3 expression (upper panel) and in *H. pylori* CagA positive and negative gastric mucosae (lower panel) (n = 65, triplicated experiments). Error bars, SD. *P* values were derived from *t* tests. n.s, not significant (*P* > 0.05); **P < 0.01; ***P < 0.001; ****P < 0.0001. (**D**) Expression of NKX6.3, p53, AICDA, and EP300 proteins in gastric cancers (n = 151) by immunohistochemistry. (**E**) Kaplan-Meier curves for overall survival of patients (n = 151) with gastric cancer with expression levels of NKX6.3, p53, EP300, and AICDA proteins. (**F**) Schematic model showing that NKX6.3 depletion in gastric epithelial cells induces mutagenesis of various genes by increasing APOBEC/AICDA activity through enhancing NFκB and CBFβ, finally resulting in the development of gastric cancer.

The presence of *H. pylori* CagA was detected in 26 (47.3%) of 55 non-neoplastic gastric mucosal tissues^14^. Compared to gastric mucosae without CagA positive *H. pylori*, gastric mucosae with *H. pylori* infection showed decreased expression of NKX6.3 but increased expression of *AICDA*, *NFκB p65*, *CBFβ*, *APOBEC3A*, and *APOBEC3B* (Fig. 6A). Interestingly, three CagA-positive *H. pylori*-infected C57BL/6 mice also showed increased mRNA expression of *AICDA*, *NFκB p65*, *APOBEC3A*, and *APOBEC3B* in gastric mucosal tissues (Fig. 6B). Thus, NKX6.3 might be able to inhibit CagA-induced mutagenesis in gastric mucosal epithelial cells.

In gastric cancers, mRNA expression levels of *AICDA, NFκB p65, CBFβ*, *APOBEC3A*, and *APOBEC3B* were markedly increased, similar to their expression profiles in gastric mucosae with *H, pylori* infection. However, *NKX6.3* expression was inversely correlated with expression of these genes (Fig. 6C). In addition, NKX6.3 mRNA expression was significantly lower in gastric cancers with higher TNM stage (Supplementary Fig. S7C), consistent with results of our previous study^15^. As expected, expression levels of *AICDA*, *NFκB p65*, *CBFβ*, *APOBEC3A*, and *APOBEC3B* were increased in gastric cancers with higher TNM stage (Supplementary Fig. S7C). We also found that mRNA expression levels of *CDH1*, *CDKN1A*, and *EP300* were significantly reduced while those of *RhoA*, *ROCK1*, *ROCK2*, *PIK3CA*, and *CCND1* were significantly increased in gastric cancers with reduced or loss of NKX6.3 expression compared to those in NKX6.3 positive cases (Fig. 6C). We also examined the presence of CagA gene in gastric cancer tissues by western blot analysis, as described previously^14^. Results showed that expression levels of *NKX6.3*, *CDH1*, *CDKN1A*, and *EP300* were decreased while expression levels of *NFκB p65*, *AICDA*, *CBFβ*, *APOBEC3A*, *APOBEC3B*, *RhoA*, *ROCK1*, *ROCK2*, *PIK3CA*, and *CCND1* were increased in CagA positive gastric cancers compared to those in CagA negative cases (Fig. 6C).

Next, we determined the relationship of NKX6.3 expression with p53, AICDA, and EP300 protein levels based on immunohistochemistry data of 151 human gastric cancer tissue specimens^15–17^. In gastric cancer tissues, NKX6.3, p53, AICDA, and EP300 proteins were expressed in 33 (21.9%), 65 (43%), 54 (35.8%), and 60 (39.7%) of 151 specimens, respectively (Fig. 6D and Table 1). There was a significant relationship between altered expression levels of NKX6.3, p53, AICDA, and EP300 proteins and clinicopathologic parameters, including depth of invasion (Chi-Square test: *P* = 0.0026, *P* = 0.2302, *P* = 0.039, and *P* = 0.0553, respectively), lymph node metastasis (Chi-Square test: *P* < 0.0001, *P* = 0.148, *P* = 0.4184, and *P* = 0.0889, respectively), and TNM stage (Chi-Square test: *P* < 0.0001, *P* = 0.1373, *P* = 0.0539, and *P* = 0.029, respectively). Importantly, NKX6.3 protein expression was positively correlated with expression of EP300 and but inversely correlated with expression of AICDA protein (Spearman’s test: *P* < 0.0001). EP300 protein expression was inversely correlated with that of AICDA protein (Spearman’s test: *P* = 0.0058) (Table 1). Kaplan-Meier analysis showed that there was no significant correlation between patient survival and p53 expression, EP300 expression, or AICDA expression alone (data not shown). However, combined analysis revealed that patients with negative NKX6.3 expression and/or negative p53, negative EP300, and positive AICDA expression had shorter overall survival time compared to those with positive NKX6.3 expression and/or positive p53, positive EP300, negative AICDA expression (*P* < 0.0001) (Fig. 6E). In 32 gastric cancers from TCGA datasets, cases with APOBEC3B and/or AICDA expression without NKX6.3 expression showed significantly higher number of mutations than those with NKX6.3 expression (Supplementary Fig. S7D). Taken together, these results indicate that NKX6.3 can regulate cell proliferation and apoptosis as well as cell motility and invasion processes by preventing accumulation of genetic alterations through downregulation of AICDA, NFκB, CBFβ, and APOBEC3B expression in gastric epithelial cells, thereby contributing to the development and progression of gastric cancer.

**Table 1:** The relationships between NKX6.3, p53, EP300 and AICDA protein expression and clinical pathological parameters in gastric cancer.

	NKX6.3		P value	p53		P value	EP300		P value	AICDA		P value
	+	−		+	−		+	−		+	−	
Total	33	118	<0.0001	65	86	0.1036	60	91	0.0146	54	97	0.0006
Tumor volume			0.9364			0.3856			0.5741			0.6175
<6.5	18	63		38	43		30	51		27	54	
≥6.5	15	55		27	43		30	40		27	43	
Gender			0.4212			0.599			0.392			0.3827
Male	25	73		40	58		41	57		38	60	
Female	8	45		25	28		19	34		16	37	
Age			0.9364			0.3856			0.5741			0.6175
≤60	20	69		34	55		37	52		31	58	
>60	13	49		31	31		23	39		23	39	
Site			0.4212			0.599			0.392			0.6237
Upper	5	10		5	10		8	7		4	11	
Lower	28	108		60	76		52	84		50	86	
L/N metastasis			0.8262			0.8059			0.7136			0.6033
LN (+)	32	116		63	85		59	89		52	96	
LN (−)	1	2		2	1		1	2		2	1	
Tumor stage			0.0026			0.2302			0.0553			0.039
T1	0	0		0	0		0	0		0	0	
T2	18	28		18	28		24	22		10	36	
T3	15	87		47	55		36	66		42	60	
T4	0	3		0	3		0	3		2	1	
N stage			<0.0001			0.148			0.0889			0.4184
N0	3	0		3	0		3	0		0	3	
N1	10	9		10	9		10	9		5	14	
N2	9	25		15	19		13	21		12	22	
N3	11	84		37	58		34	61		37	58	
TNM stage			<0.0001			0.1373			0.029			0.0539
I	1	0		1	0		1	0		0	1	
II	17	9		15	11		16	10		5	21	
III	15	107		49	73		43	79		47	75	
IV	0	2		0	2		0	2		2	0	
Lauren’s			0.3217			0.5166			0.324			0.3694
intestinal	11	53		30	34		22	42		26	38	
diffuse	22	65		35	52		38	49		28	59	
p53			0.6066									
+	16	49										
−	17	69										
EP300			<0.0001			0.8214						
+	25	35		27	33							
−	8	85		38	53							
AICDA			<0.0001			0.4394			0.0056			
+	0	54		26	28		13	41				
−	33	64		39	58		47	50				

## Discussion

Recently, several studies have revealed genetic alterations underlying gastric carcinogenesis by multi-omics profiling^3–5,18^. A mutational signature highlights specific carcinogenic and mutation processes in gastric cancer, including microsatellite instability, CpG associated deamination, and activation of cytidine deaminases such as AICDA and APOBEC3B^3,4,7,18^. Here, we observed that NKX6.3 depletion could lead to somatic mutations, mainly G or C to A or T base pair transitions in coding regions in gastric epithelial cells, consistent with results of TCGA-based analysis (Fig. 1A and Supplementary Fig. 1). For example, NKX6.3 depletion induced some driver mutations in *CDH1*, *TP53*, *RhoA*, *PIK3CA*, and *EP300* genes, consistent with previous findings in gastric cancer^3–5^. CDH1 acts as a tumor suppressor. Decreased expression of CDH1 has been observed in gastric cancer with somatic mutation and methylation in the gene^19,20^. TP53 is another important tumor suppressor that negatively regulates cell cycle. Aberrant activity of TP53 caused by mutations is required for tumor formation^21^. Loss of p53 function can lead to impaired DNA replication, malignant transformation, genetic instability, and increased survival of cells with increased mutational load^22^. RhoA is a key member of the Rho family of small GTP-binding proteins that act as mediators between cell surface receptors and different intracellular signaling proteins^23^. RhoA overexpression has been observed in various cancers and RhoA activity has been implicated in tumorigenesis and tumor cell invasion^24^. In addition, highly recurrent mutations of *RhoA* have been detected in diffuse-type of gastric cancers^5,25^. Mutations in *PIK3CA* which encodes p110 catalytic subunit of PI3K have been observed in various human cancers, including gastric cancers. These mutations can lead to activation of PI3K/AKT and its downstream signaling pathways, thereby contributing to carcinogenesis^26–28^. EP300 functions as a histone acetyltransferase. It is an important modulator in cell proliferation and differentiation through transcriptional regulation via chromatin remodeling^29,30^. Loss of EP300 by inactivating mutations may contribute to tumorigenesis in human cancers, including gastric cancers^31^. Caspase-8 is activated by its recruitment to Fas-mediated apoptosis^32^. Somatic mutations of caspase-8 can lead to attenuation of its proapoptotic function and contribute to gastric carcinogenesis^33^. Here, we found that depletion of NKX6.3 could drive tumorigenesis in xenograft mouse model while tumor associated genes including p53, PI3K, E-cadherin, and EP300 were aberrantly expressed in these tumors. Consistent with results of NKX6.3 depleted cells, xenograft tumor also showed mutations in these genes (Figs 1, 4 and 5). Thus, NKX6.3 depletion might lead to multiple genetic mutations known to be closely associated with gastric tumorigenesis.

APOBECs and AICDA can mutate a host’s DNA. Significant numbers of APOBEC and AICDA-induced mutations have been observed in many types of human cancers^34–38^. Gastric cancer cells are highly enriched in mutation signatures [TC(A|T) → (T|G)] and [(A|T)(A|G)C → (T|G)] that are characteristic of a subclass of APOBEC and AICDA cytidine deaminases^39–41^. Two members of the APOBEC family, APOBEC3A and APOBEC3B, contribute substantially to mutations in cancers by deaminating cytosines in the TpCpW context^34–37,40,41^. It has been found that TpCpW mutations occur more frequently in gastric cancers with APOBEC expression^38^. APOBEC3B could activate genome-wide mutagenesis in various cancers^34–37,40^. In addition, aberrant expression of AICDA via NFκB activation causes accumulation of mutation in gastric epithelium with *H. pylori* infection^7^. AICDA-induced mutagenesis can lead to genome-wide alterations in the known preferred AICDA target sequence such as WRC motifs^39,42^. Moreover, CBFβ is required for *APOBEC3* gene expression while NFκB is required for AICDA gene expression^7,13^. In the present study, we found that expression of *CBFβ* and *NFκB* was significantly increased in gastric cancer tissues while knockdown of *CBFβ* or *NFκB* dramatically inhibited the expression of APOBEC family and AICDA genes, diminished deaminase activity, and reduced the frequency of NKX6.3 depletion-induced mutations in *TP53*, *PIK3CA*, and *POLA1* genes. Notably, binding of NKX6.3 to the promoter region of *CBFβ* and *NFκB* downregulated their expression while depletion of NKX6.3 dramatically increased expression of APOBEC gene family and AICDA genes at mRNA and protein levels, suggesting that NKX6.3 might function as a transcriptional repressor of these genes and downregulate deaminase activity in gastric epithelial cells (Fig. 2). Thus, NKX6.3 might inhibit the APOBEC- and AICDA-induced mutations by acting as a transcriptional repressor of *APOBEC family* and *AICDA* genes, thereby protecting gastric epithelial cells against genome-wide genetic mutations.

In non-neoplastic gastric mucosae and gastric cancer tissues, expression levels of *CBFβ*, *NFκB p65*, *APOBEC family*, and *AICDA* were increased in the cases with reduced or loss of NKX6.3 expression while NKX6.3 expression was inversely correlated with expression of these genes. In addition, expression levels of *CDH1*, *CDKN1A*, and *EP300* were reduced while expression levels of *RhoA*, *ROCK1*, *ROCK2*, *PIK3CA*, and *CCND1* were increased in gastric cancer tissues with reduced or loss of NKX6.3 expression (Fig. 6). Previously, we have reported that NKX6.3 expression is involved in gastric cancer progression and patients’ survival^15^. Concordant with these results, gastric cancer patients with reduced or loss of NKX6.3 expression and/or high mutation rates had shorter overall survival time (Supplementary Fig. S8E). In immunohistochemistry analysis, NKX6.3 expression was inversely associated with AICDA expression but positively associated with EP300 expression in 151 gastric cancer tissues (Fig. 6D). Thus, patients with reduced or loss of NKX6.3 expression and/or negative p53, negative EP300, and positive AICDA expression had shorter overall survival time (Fig. 6E), providing strong support for our hypothesis.

In conclusion, our study provides a new paradigm for the role of NKX6.3 in the pathogenesis of gastric cancer. We demonstrated that NKX6.3 depletion could lead to aberrant expression of AICDA and APOBEC family genes in gastric epithelial cells, thereby generating widespread genetic mutations and eventually driving the development and progression of gastric cancer (Fig. 6F). These findings unambiguously indicate that depletion of NKX6.3 in gastric epithelial cells can prompt accumulation of genetic mutations, providing important genetic evidence for the molecular mechanism involved in the development of gastric cancers.

## Materials and Methods

### Human Gastric Samples

A total of 65 frozen gastric cancers were obtained from Chonnam National University Hwasun Hospital supported by the Ministry of Health, Welfare and Family Affairs. In addition, a total of 55 patients with sporadic gastric cancer who underwent gastrectomy at Seoul St. Mary’s Hospital were included. Fifty-five non-neoplastic gastric mucosae remote from the tumor (>5 cm) at each corresponding region were enrolled in this study. Informed consent was provided according to the Declaration of Helsinki. Written informed consent was obtained from all subjects. This study was approved by the Institutional Review Board of The Catholic University of Korea, College of Medicine (approval numer: MC16SISI0130). There was no evidence of familial cancer in any of these patients.

For immunohistochemical analysis, tissue microarray recipient blocks were constructed, containing 151 gastric cancer tissues from formalin-fixed paraffin embedded specimens at Seoul St. Mary’s Hospital. Three tissue cores from each cancer (2 mm in diameter) were taken and placed in a new recipient paraffin block using a commercially available microarray instrument (Beecher Instruments, Micro-Array Technologies, Silver Spring, MD, USA) according to established methods^15^. One cylinder of normal gastric mucosa adjacent to each tumor was also transferred to the recipient block. Sections were cut in thickness of 2 μm the day before use. They were stained according to standard protocols.

Other details regarding cell culture, transfection, whole genome sequencing, gene set enrichment analysis, detecting AICDA/APOBEC mutation pattern, *in vitro* deaminase assay, 3D-PCR, cloning, sequencing, validation detection, caspase 3/7 activity assay, motility and invasion assays, spheroid cell culture, *in vivo* xenograft mouse experiment, RT–qPCR and ChIP–qPCR, immunoblotting and immunofluorescence (IF), immunohistochemistry (IHC), construction of plasmids, transient reporter assay, bacterial strain and animal infection, datasets, and statistical analyses are available in Supplementary Materials and Methods.

## Electronic supplementary material

Supplementary Information

Supplementary Table S2

## References

[1] Hu B (2012). Gastric cancer: Classification, histology and application of molecular pathology. J Gastrointest Oncol.

[2] Ferlay J (2015). Cancer incidence and mortality worldwide: sources, methods and major patterns in GLOBOCAN 2012. Int J Cancer.

[3] Wang K (2011). Exome sequencing identifies frequent mutation of ARID1A in molecular subtypes of gastric cancer. Nat Genet.

[4] Bass AJ (2014). Comprehensive molecular characterization of gastric adenocarcinoma. Nature.

[5] Wang K (2014). Whole-genome sequencing and comprehensive molecular profiling identify new driver mutations in gastric cancer. Nat Genet.

[6] Touati E (2003). Chronic Helicobacter pylori infections induce gastric mutations in mice. Gastroenterology.

[7] Matsumoto Y (2007). Helicobacter pylori infection triggers aberrant expression of activation-induced cytidine deaminase in gastric epithelium. Nat Med.

[8] Conticello SG (2008). The AICDA/APOBEC family of nucleic acid mutators. Genome Biol.

[9] Alanentalo T (2006). Cloning and analysis of Nkx6.3 during CNS and gastrointestinal development. Gene Expr Patterns.

[10] Choi MY (2008). Requirement of the tissue-restricted homeodomain transcription factor Nkx6.3 in differentiation of gastrin-producing G cells in the stomach antrum. Mol Cell Biol.

[11] Yoon JH (2016). Inactivation of NKX6.3 in stomach leads to abnormal expression of CDX2 and SOX2 required for gastric-to-intestinal transdifferentiation. Mod Pathol.

[12] Yoon JH (2015). NKX6.3 controls gastric differentiation and tumorigenesis. Oncotarget.

[13] Anderson BD, Harris RS (2015). Transcriptional regulation of APOBEC3 antiviral immunity through the CBF-β/RUNX axis. Sci Adv.

[14] Yoon JH (2014). Gastrokine 1 inhibits the carcinogenic potentials of Helicobacter pylori CagA. Carcinogenesis.

[15] Yoon JH (2016). NKX6.3 Is a Transcription Factor for Wnt/β-catenin and Rho-GTPase Signaling-Related Genes to Suppress Gastric Cancer Progression. EBioMedicine.

[16] Kim CJ (2007). Activation-induced cytidine deaminase expression in gastric cancer. Tumour Biol.

[17] Eun JW (2017). MicroRNA-495-3p functions as a tumour suppressor by regulating multiple epigenetic modifiers in gastric carcinogenesis. J Pathol.

[18] Wong SS (2014). Genomic landscape and genetic heterogeneity in gastric adenocarcinoma revealed by whole-genome sequencing. Nat Commun.

[19] Guilford P (1998). E-cadherin germline mutations in familial gastric cancer. Nature.

[20] Strathdee G (2002). Epigenetic versus genetic alterations in the inactivation of E-cadherin. Semin. Cancer Biol.

[21] Weisz L, Oren M, Rotter V (2007). Transcription regulation by mutant p53. Oncogene.

[22] Yeo CQ (2016). p53 Maintains Genomic Stability by Preventing Interference between Transcription and Replication. Cell Rep.

[23] Ridley AJ (2003). Cell migration: integrating signals from front to back. Science.

[24] Sahai E, Marshall CJ (2002). RHO-GTPases and cancer. Nat Rev Cancer.

[25] Zhou J, Hayakawa Y, Wang TC, Bass AJ (2014). RhoA mutations identified in diffuse gastric cancer. Cancer Cell.

[26] Samuels Y (2004). High frequency of mutations of the PIK3CA gene in human cancers. Science.

[27] Samuels Y (2005). Mutant PIK3CA promotes cell growth and invasion of human cancer cells. Cancer Cell.

[28] Shigaki H (2013). PIK3CA mutation is associated with a favorable prognosis among patients with curatively resected esophageal squamous cell carcinoma. Clin Cancer Res.

[29] Kawasaki H (1998). Distinct roles of the co-activators p300 and CBP in retinoic-acid-induced F9-cell differentiation. Nature.

[30] Yao TP (1998). Gene dosage-dependent embryonic development and proliferation defects in mice lacking the transcriptional integrator p300. Cell.

[31] Gayther SA (2000). Mutations truncating the EP300 acetylase in human cancers. Nat Genet.

[32] Nagata S (1997). Apoptosis by death factor. Cell.

[33] Soung YH (2005). CASPASE-8 gene is inactivated by somatic mutations in gastric carcinomas. Cancer Res.

[34] Burns MB, Temiz NA, Harris RS (2013). Evidence for APOBEC3B mutagenesis in multiple human cancers. Nat Genet.

[35] Burns MB (2013). APOBEC3B is an enzymatic source of mutation in breast cancer. Nature.

[36] Chan K (2015). An APOBEC3A hypermutation signature is distinguishable from the signature of background mutagenesis by APOBEC3B in human cancers. Nat Genet.

[37] Seplyarskiy VB (2016). APOBEC-induced mutations in human cancers are strongly enriched on the lagging DNA strand during replication. Genome Res.

[38] Alexandrov LB (2013). Signatures of mutational processes in human cancer. Nature.

[39] Rogozin IB (2016). Activation induced deaminase mutational signature overlaps with CpG methylation sites in follicular lymphoma and other cancers. Sci Rep.

[40] Kuong KJ, Loeb LA (2013). APOBEC3B mutagenesis in cancer. Nat Genet.

[41] Roberts SA (2013). An APOBEC cytidine deaminase mutagenesis pattern is widespread in human cancers. Nat Genet.

[42] Matsumoto T, Shimizu T, Takai A, Marusawa H (2015). Exploring the Mechanisms of Gastrointestinal Cancer Development Using Deep Sequencing Analysis. Cancers.

