# Supplementary material for: Multiple genetic mutations caused by NKX6.3 depletion contribute to gastric tumorigenesis
Source: Sci Rep. 2018 Dec 4;8:17609. doi: 10.1038/s41598-018-35733-5 (PMC6279800; doi:10.1038/s41598-018-35733-5)
Supplement: Supplementary file 1 — Supplementary Information [file 41598_2018_35733_MOESM1_ESM.pdf]

Multiple genetic mutations caused by NKX6.3 depletion contribute to gastric tumorigenesis

Jung Hwan Yoon, Olga Kim, Jung Woo Eun, Sung Sook Choi, Hassan Ashktorab, Duane T. Smoot, Suk Woo Nam, Won Sang Park

Supplementary Materials and Methods

Cell Culture and Transfection

AGS and MKN1 gastric cancer cells lines and an HFE-145 immortalized non-neoplastic gastric epithelial cell lines[1] were cultured at 37°C in 5% CO_2 in RPMI-1640 and DMEM medium with 10% heat-inactivated fetal bovine serum. Complete *NKX6.3*-cDNA was cloned into the expression vector pCMV6-Myc-DDK (Origene, Rockville, MD, USA) and shNKX6.3 was cloned into the pGFP-C-shLenti vector (Origene). AGS and MKN1 cells were transiently transfected with expression plasmids (5 μg total DNA) and the HFE-145 cells were transiently transfected with *shNKX6.3*, *siNFκB*, *siCBFβ*, *siAPOBEC3B* and *siAICDA* in 60 mm-diameter dishes using Lipofectamine Plus transfection reagent (Invitrogen), according to the manufacturer's recommendations.

We generated stable NKX6.3 transfectants of the AGS and MKN1 cells, $AGS^{NKX6.3}$ and $MKN1^{NKX6.3}$, stably expressing human NKX6.3, as well as mock transfectants, AGS^{Mock} and $MKN1^{Mock}$ cells. We also generated stably NKX6.3 knockdowned cells in two different target sites, $HFE\text{-}145^{shNKX6.3\#1}$ and $HFE\text{-}145^{shNKX6.3\#2}$, as well as non-targeting shRNA transfectants, $HFE\text{-}145sh^{Ctrl}$ cells, described previously[2]. Stable expression or knockdown of NKX6.3 was confirmed in the $AGS^{NKX6.3}$, $MKN1^{NKX6.3}$ and $HFE\text{-}145^{shNKX6.3}$ cells by western blot analysis.

Whole Genome Sequencing

The samples were prepared according to the Illumina TruSeq Nano DNA library preparation guide or TruSeq DNA PCR-free library preparation guide. Briefly, 0.5–3 μg

of DNA from the HFE-145^{shCtrl}, HFE-145$^{shNKX6.3\#1}$ and HFE-145$^{shNKX6.3\#2}$ cells were used to prepare the sequencing library by shearing the DNA followed by ligating the sequencing adaptors. Captured DNA was sequenced using the Illumina HiSeq X Ten platform and generated paired-end sequencing reads for the HFE-145^{shCtrl}, HFE-145$^{shNKX6.3\#1}$ and HFE-145$^{shNKX6.3\#2}$ cells. We checked the quality of the sequence reads using FastQC 0.1 software. Paired-end libraries of approximately 500-bp inserts were sequenced as 150-bp paired-end reads. Raw sequencing data were processed by an ultrafast Isaac DNA sequence aligner designed to align next-generation sequencing data with low-error rates using human genome hg19, GRCh37. The Isaac Variant Caller was used to call single-nucleotide variants (SNVs) and small indels from the bam files[3]. SnpEff was used for annotating the variants with dbSNP138, dbSNP142, 1000 Genomes project and ESP6500 database[4]. We excluded common single-nucleotide polymorphisms (SNPs) with minor allele frequency of > 0.001 as recorded in either dbSNP.

Gene Set Enrichment Analysis

Gene sets were downloaded from the FunRich: Functional Enrichment analysis tool and Gene set permutations[5] were used to determine the statistical enrichment of the gene sets using the signal-to-noise ratios of NKX6.3 depletion-induced mutated genes.

Detecting an AICDA/APOBEC mutation pattern

The numeric value of enrichment E that characterized the strength of mutagenesis at the tCw and wrC motif in mutation clusters was calculated as

$$E = \frac{\text{mutations(tCw or wrC)} \times \text{context (c or g)}}{\text{mutations(C or G)} \times \text{context (tcw or wrc)}}$$

Where mutations tCw or wrC are the number of mutated C or G that fall in a tC̲w or wG̲a and wrC̲ or G̲yw motifs, mutations C or G is the total number of mutated C or G, context tcw or wrc is the total number of tcw or wga and wrc or gyw motifs within the 21-nucleotides region centered on the mutated C or G and context c or g is the total number of c or g within the 21-nucleotides region centered on the mutated C or G. The enrichment of the APOBEC and AICDA mutation pattern was calculated as above by only specific base substitutions (tCw to tTw or tGw, wGa to wAa or wCa, wrC to wrT or wrG, Gyw to Ayw or Cyw, C to T or G, and G to A or C), as previously described[6].

FRET-based in vitro Deamination Assay

A fluorescence resonance energy transfer (FRET)-based assay were performed previously described[7]. The cells were extensively washed with PBS and mechanically harvested them. The total proteins were extracted using specific lysis buffer (25 mM HEPES [pH 7.4], 10% glycerol, 150 mM NaCl, 0.5% Triton X-100, 1 mM EDTA, 1 mM $MgCl_2$ and 1 mM $ZnCl_2$) supplemented with protease inhibitors and submitted then to sonication. Deaminase activity was assessed by incubating whole-cell lystates with 1 pmol DNA oligonucleotide 5′-(6-FAM)-AAATTCTAATAGATAATGTGA-(TAMRA)-3′ in the presence of 0.4 unit uracil-DNA glycosylase (NEB) in a 20 mM Tris-HCl, 1 mM dithiothreitol, 1 mM EDTA reaction buffer. After 2 h incubation at 37 °C, the generated abasic sites were cleaved by heating 2 min at 95 °C and measured end point fluorescence using the Bio-Rad IQ5 real-time PCR platform (Bio-Rad) with FAM setting and

background fluorescence obtained with mock-transfected cells as a negative control. The results are normalized to the quantity of protein using the Pierce BCA Protein Assay Kit (Thermo Scientific).

3D-PCR, Cloning, and Sequencing

TP53 3D-PCR was performed by modifying a previously published method[8,9], performing the initial PCR as follows: 94°C for 5 min, followed by 35 cycles, each at 94°C for 20 s, 65°C for 30 s, and 72°C for 30 s, and a final elongation step at 72°C for 10 min. Nested PCR was performed as follows: 92 to 82°C for 5 min, followed by 35 cycles, each at 92 to 82°C for 30 s, 60°C for 30 s, and 72°C for 30 s, and a final elongation step at 72°C for 10 min on a MasterCycler Pro thermal cycler (Eppendorf, Seoul, Korea). To determine the hypermutation frequency, the PCR fragments from 3D-PCR or standard PCR (94°C denaturation) were cloned into a TOPO-TA cloning vector (Invitrogen) and then randomly selected the indicated number of successful recombinant clones and sequenced them using an ABI Prism 3130 (Applied Biosystems, Foster City, CA, USA).

Validation Detection

To confirm genetic mutation for PIK3CA, TP53, CDH1, RhoA, EP300, Casp8 and POLA1 genes obtained by whole genome sequencing using PCR amplification and Sanger sequencing of cDNA. The PCR reactions were 10 min at 95ºC; 35 cycles of 30 sec at 95ºC, 30 sec at 55-65ºC, and 30 sec at 72ºC, and, finally, 10 min at 72ºC. PCR and Sanger sequencing primers were described in Star resource table. All the Sanger sequencing experiments were performed at Bionics Inc. (http://www.bionicsro.co.kr).

Caspase 3/7 Activity Assay

The HFE-145^{shCtrl} and HFE-145$^{shNKX6.3}$ cells with/without anti-Fas were homogenized in lyses buffer, and caspase activities were measured using Caspase-Glo 3/7 assay kit according to the manufacturer's instruction (Promega, Madison, WI). In 96-well plates, a 50-μl sample was mixed gently for 30 s with 50 μl Caspase-Glo 3/7 reagent and incubated for 2 h at room temperature. The lyses buffer with the reagent served as blank. Luminescence of the samples was measured using an Infinite M200 plate reader (Tecan, Research Triangle Park, NC). Luminescent intensity values were normalized to the sample protein concentration.

Motility and Invasion Assays

For *in vitro* cell motility and invasion assays, transwell plates and cell culture inserts (BD Biosciences) were used. For the coating of the invasion assay, Matrigel (BD Biosciences) was diluted to 0.3 mg/ml concentration with coating buffer (0.01 M Tris, 0.7% NaCl, pH 8.0) and 100 μl Matrigel was coated onto the upper compartment of the cell culture insert. After incubation for 1 h at 37°C, the cell culture insert was ready for seeding. HFE-145^{shCtrl} and HFE-145$^{shNKX6.3}$ cells were appropriately seeded (0.5×10^4 cells/well for the motility assay, 1×10^4 cells/well for the invasion assay) into the cell culture insert with serum-free medium in the presence of 2% FBS as chemoattractant. After incubation for 12 h (migration assay) or 24 h (invasion assay) at 37°C, migrated or invaded cells were stained using Diff-Quik staining kit (Sysmex, Japan). Cells were photographed using an Axiovert 200 inverted microscope (Zeiss, Jena, Germany) at x200 magnification. Cells were enumerated in three random fields of view.

Spheroid Cell Culture

For growing HFE-145 spheroid cells, we used DMEM/F12 (Catalog no. 12500-062; Gibco) medium optimized for growth of neural stem cells, conditioned with glucose 1.55 g/L (Sigma-Aldrich, St. Louis, MO, USA); L-Glutamine 73 mg/L (Sigma-Aldrich); sodium bicarbonate 1.69 g/L (Sigma-Aldrich); N-2 Plus Media Supplement ×1000 containing bovine insulin, human transferrin, sodium selenite, putrescine, and progesterone (R&D Systems, Minneapolis, MN, USA); FGF basic 100 ng/mL; EGF 100 ng/mL (both from R&D Systems); penicillin 100 U/mL (Gibco-Invitrogen); and streptomycin 100 μg/mL (Gibco-Invitrogen). Daily supplementations were added with EGF 100 ng/mL and FGF basic 100 ng/mL. Cell morphology was examined under light microscopy (Olympus IX70; Center Valley, PA, USA) and photographed using a Nikon Coolpix 5000 digital camera (Melville, NY, USA).

In vivo Xenograft Mouse Experiment

For in vivo xenograft assay, 5 x 10^6 cells of HFE-145^{shCtrl}, HFE-$145^{shNKX6.3\#1}$ and HFE-$145^{shNKX6.3\#2}$ cells were mixed with 0.2 ml PBS (pH7.4) and 30% (v/v) Matrigel (BD Biosciences), respectively. The BALB/c-nude mice between 4 and 5 weeks were purchased from ORIENT (Seongnam, Korea). The cells were inoculated subcutaneously into side of flank of the nude mouse. Mice were examined twice per week for tumor formation at the injection site. After three weeks, the tumor volumes were measured every three days and calculated as length×width^2×0.5. After 7 weeks of tumor formation, the

tumors were carefully removed, photographed and weighed. Each experimental group consisted of 4 mice and tumor growth was quantified by measuring tumor sizes using calipers. All animal experiments were undertaken in according to the National Institutes of Health's Guide for the Care and Use of Laboratory Animal, with approval of the Animal Experiment Ethics Committee of the Catholic University of Korea College of Medicine (CUMC-2017-0018-01).

RT–qPCR and ChIP–qPCR

RNA was isolated from cells using the RNeasy kit (Qiagen, Valencia, CA, USA) and then reverse transcribed it to cDNA (Life Technologies, Carlsbad, CA, USA), then qPCR was performed for quantification using standard procedures on a Bio-Rad IQ5 real-time PCR platform (Bio-Rad Laboratories, Hercules, CA, USA). To ensure the fidelity of mRNA extraction and reverse transcription, all samples were subjected to PCR amplification with oligonucleotide primers specific for the constitutively expressed gene, *glyceraldehyde-3-phosphate dehydrogenase (GAPDH)* and normalized. The mean value of mRNA expression in gastric mucosae without *H. pylori* infection was used as a control. mRNA expression change in each case was further normalized to mean value of those in control gastric mucosae and reduced mRNA expression was defined less than 0.5-fold change. According to the negative NKX6.3 expression was defined less than 0.5-fold change, compared non-neoplastic gastric mucosae.

ChIP was performed as previously described[1] with modifications. In brief, cells were cross-linked in 1% formaldehyde (Thermo Scientific, Wilmington, DW, USA) in PBS for 10 min at room temperature. After glycine quenching, the cell pellets were collected and

lysed as previously described[1] and then subjected them to sonication with the Covaris S220 sonicator (Covaris, Woburn, MA, USA). The supernatant was then diluted in the same sonication buffer without N-lauroylsarcosine and subjected it to immunoprecipitation with corresponding antibodies at 4°C overnight. The beads were then washed and DNA was reverse-cross-linked and purified. Following ChIP, the DNA was quantified by qPCR using standard procedures on the Bio-Rad IQ5 real-time PCR platform (Bio-Rad). All primers described in Supplementary Table 7.

Immunoblotting and Immunofluorescence (IF)

The effects of NKX6.3 on expression of NFκB, CBFβ, AICDA/APOBEC family and mutant genes including NFκB p65, CBFβ, AICDA and APOBEC3B were determined in HFE-145^{shCtrl}, HFE-145$^{shNKX6.3}$ and xenograft tumor tissues by immunoblot, immunofluorescence and confocal microscopy, as previously described[10]. Briefly, the cells and mouse tissues were washed twice with cold PBS and lysed in RIPA buffer that contained 50 mM Tris pH 7.4, 150 mM NaCl, 1% NP-40, 0.5% sodium deoxycholate, 0.1% SDS, 1 mM phenylmethyl sulfonyl fluoride, complete protease, and phosphatase inhibitor cocktail (Roche, Indianapolis, IN, USA) for 5 min and collected them by scraping. The lysates were cleared by centrifuging at 12,000 rpm for 10 min. Protein was quantified by BCA Assay and separated the sample proteins by SDS–PAGE gel electrophoresis. Next, the proteins were transferred from gel to a PVDF membrane (Bio-Rad). After that, membranes were blocked in 5% BSA-TBST for 30 min at room temperature, and then incubated them overnight with indicated primary antibodies at 4 °C. After that, the membranes were washed three times,

incubated them with peroxidase-conjugated secondary antibodies (Sigma, St. Louis, MD, USA) and detected them with enhanced chemiluminescence (Millipore, Billerica, MA, USA) on LAS-4000.

The effect of NKX6.3 on protein expression of mutant genes was determined in xenograft mouse tissues by immunofluorescence and confocal microscopy, as previously described[10]. In brief, xenograft tumor tissue blocks were constructed from formalin-fixed paraffin embedded specimens, as previously described[10]. 2 μm sections were cut the day before use and stained according to standard protocols. The tissues were then incubated with a rabbit anti-NKX6.3 (protein atlas), CBFβ, APOBEC3B, RhoA, p53, PI3K, p-Akt (cell signaling) polyclonal antibody, and mouse anti-NFκB p65, AICDA, E-cadherin, p21, and EP300 (cell signaling) monoclonal antibody in PBS containing 0.5% Triton X-100 overnight. The tissue slides were rinsed with PBS and incubated with an Alexa-488 conjugated goat anti-rabbit IgG (invitrogen) or incubated with a Texas-Red conjugated goat anti-mouse IgG (invitrogen) for 1 hr at room temperature. Counterstaining of cell nuclei was carried out by incubating the tissues with DAPI (4',6-diamidino–2'-phenylindole; Roche; dilution 1:1000) for 10 min. Slides were viewed with a confocal microscope (LSM 510 Meta, Carl Zeiss Co., Ltd., Germany). Images were converted to TIFF format, and contrast levels were adjusted using Adobe Photoshop v. 7.0 (Adobe Systems, San Jose, CA, USA). All antibodies described in Supplementary Table 8.

Immunohistochemistry (IHC)

In order to analyze the relationship between expression of NKX6.3, TP53, AICDA, and EP300 and clinicopathological parameters in 151 gastric cancer tissues, we obtained

immunohistochemical data from our previously studies[10-12].

Construction of Plasmids

Promoter regions of the *APOBEC family*, *Ung*, *Apex1* and *CBFβ* genes, which contain putative NKX6.3 binding elements, were amplified by PCR of genomic clones that contained *APOBEC family*, *Ung*, *Apex1* and *CBFβ*. All fragments were digested with KpnI and XhoI and were cloned into the pGL3-Luciferase reporter basic vector (Promega, Madison, WI, USA) upstream of the firefly luciferase–encoding region.

Transient Reporter Assay

Equimolar amounts of reporter plasmids were transiently transfected into AGS^{Mock}, $AGS^{NKX6.3}$, $MKN1^{Mock}$, $MKN1^{NKX6.3}$, HFE-145^{shCtrl} and HFE-$145^{shNKX6.3}$ cells by lipofectamine, as described previously[10]. The general transfection efficiency of this procedure was more than 75%, as monitored by a reporter plasmid encoding green fluorescent protein. Each transfection included 50 ng phRL (renilla luciferase expression construct; Promega), driven by the CMV immediate early promoter, as an internal control. The cells were cultured for 24 h with or without stimulation before being collected. The luciferase activity was measured with the Dual-Luciferase Assay kit (Promega) with an EnVision 2103 Multilabel Reader (PerkinElmer, Boston, MA, USA) and normalized the firefly luciferase activity to renilla luciferase activity and was presented as the 'fold change' relative to that obtained with pGL3-Luciferase reporter control vector (Promega).

Bacterial Strain and Animal Infection

To construct the *CagA* knockout mutant, *H. pylori* 26695 (reference strain, $CagA^{+}$, $vacA^{+}$) was used, as previously described[13]. The *H. pylori* was cultured at 37°C in a standard microaerobic atmosphere (5% O_2, 10% CO_2, and 85% N_2) in brain–heart infusion (BHI) medium (Difco, Detroit, MI, USA) with 7% laked horse blood (Oxoid, Cambridge, UK), 0.4% IsoVitalex™ (BBL, Sparks, MD, USA), vancomycin (6 μg/ml), amphotericin B (8 μg/ml), and trimethoprim (5 μg/ml). Five C57BL/6 female mice aged 5 weeks were purchased from *Qu*-BEST (Seongnam, Korea) and inoculated three mice three times by oral gavage with 0.4 ml of the suspension containing *H. pylori* 26695 ($2x10^9$ c.f.u. ml^{-1}). Four weeks post-inoculation, two controls and three mice were sacrificed, and their gastric mucosal tissues were used for molecular studies and to determine colonization.

Datasets

To determine whether levels of NKX6.3 correlated with mutation rate in larger gastric cancer cohort, we obtained whole-genome-seq- and RNA-seq-based gene mutations and expression data from The Cancer Genome Atlas (TCGA) stomach adenocarcinoma project. The RNA-seq data were analyzed by first replacing all RSEM values that equaled zero with the smallest nonzero RSEM value and then applied a log2 transformation. According to the mean expression value of NKX6.3 in 395 gastric cancers, the samples were divided into high and low expression groups.

Statistical analysis

Student's t-test was used to analyze the expression of NKX6.3 in the gastric cancer tissues and cell lines as well as ChIP and mRNA expression. Pearson's and linear regression tests were used to analyze the expression of NKX6.3, NFκB, CBFβ and AICDA/APOBEC family in gastric cancer tissues. Chi-square and Spearman's tests were used to analyze the relationship between NKX6.3, TP53, AICDA, EP300 and clinicopathological parameter in gastric cancer tissues. The Kaplan-Meier method was used for analysis of survival curves, and statistical significance was assessed using the log-rank test. Data are expressed as mean ± SEM from at least three independent experiments. A p-value less than 0.05 was considered to be the limit of statistical significance. All experiments were performed in triplicate to verify the reproducibility of the findings.

Supplementary references

1. Smoot DT et al., Human gastric epithelial cell lines derived from primary cultures of normal gastric epithelial cells (Abstract). *Gastroenterology* **118**, A540 (2000).

2. Yoon JH, et al. NKX6.3 controls gastric differentiation and tumorigenesis. *Oncotarget* **6,** 28425-28439 (2015).

3. Raczy C, et al. Isaac: ultra-fast whole-genome secondary analysis on Illumina sequencing platforms. *Bioinformatics* **29,** 2041-2043 (2013).

4. Cingolani P, et al. A program for annotating and predicting the effects of single nucleotide polymorphisms, SnpEff: SNPs in the genome of Drosophila melanogaster strain w1118; iso-2; iso-3. *Fly (Austin)* **6,** 80-92 (2012).

5. Pathan M, et al. FunRich: an open access standalone functional enrichment and interaction network analysis tool. *Proteomics* **15,** 2597–2601 (2015).

6. Roberts SA, et al. An APOBEC cytidine deaminase mutagenesis pattern is widespread in human cancers. *Nat Genet* **45,** 970-976 (2013).

7. Stenglein MD, et al. APOBEC3 proteins mediate the clearance of foreign DNA from human cells. *Nat Struct Mol Biol* **17,** 222-229 (2010).

8. Suspene R, Guétard D, Henry M, Sommer P, Wain-Hobson S, Vartanian JP. Extensive editing of both hepatitis B virus DNA strands by APOBEC3 cytidine deaminases in vitro and in vivo. *Proc Natl Acad Sci USA* **102,** 8321-8326 (2005).

9. Bonvin M, et al. Interferon-inducible expression of APOBEC3 editing enzymes in human hepatocytes and inhibition of hepatitis B virus replication. *Hepatology* **43,**

1364-1374 (2006).

10. Yoon JH, et al. NKX6.3 Is a Transcription Factor for Wnt/β-catenin and Rho-GTPase Signaling-Related Genes to Suppress Gastric Cancer Progression. *EBioMedicine* **9,** 97-109 (2016).

11. Kim CJ, et al. Activation-induced cytidine deaminase expression in gastric cancer. *Tumour Biol* **28,** 333-339 (2007).

12. Eun JW, et al. MicroRNA-495-3p functions as a tumour suppressor by regulating multiple epigenetic modifiers in gastric carcinogenesis. *J Pathol* **244,** 107-119 (2017).

13. Yoon JH, et al. Gastrokine 1 inhibits the carcinogenic potentials of Helicobacter pylori CagA. *Carcinogenesis* **35,** 2619-2629 (2014).

Supplementary Table S1. Summary of whole-genome sequencing.

(**A**) Summary of Pre- and Post-alignment Statistics.

Pre-alignment Statistics

	shCtrl	shNKX6.3[#1]	shNKX6.3[#2]
**Total Number of reads**	969,434,140	1,012,493,600	946,834,370
**Read length (bp)**	150.00	150.00	150.00
**Total yield (Mbp)**	145,415	151,874	142,025
**Reference size (Mbp)**	2,858	2,858	2,858
**Throughput mean depth (X)**	50.90	53.10	49.70

*Total yield: (total number of reads) x (read length)

*Reference size: Non-N human genome reference size

*Throughput mean depth: (total yield) / (reference size)

Post-alignment Statistics

	shCtrl	shNKX6.3[#1]	shNKX6.3[#2]
**De-duplicated reads**	899,862,436	947,490,328	888,254,572
**De-duplicated reads %**	92.80	93.60	93.80
**Mappable reads (reads mapped to human genome)**	801,781,769	854,357,077	759,787,548
**Mappable reads % (out of de-duplicated reads)**	89.10	90.20	85.50
**Mappable yield (Mbp)**	120,267	128,153	113,968
**Mappable mean depth (X)**	42.10	44.80	39.90

*Non-N human genome reference size: 2,858 Mbp

*De-duplicated reads %: 100 x (number of de-duplicated reads) / (total number of reads)

Mappable reads %: 100 x (number of mappablr reads) / (number of de-duplicated reads)

*Mappable yield: (number of mappable reads) x (read length)

*Mappable mean depth (X): (mappable yield) / (referece size)

(**B**) Summary of algnment coverage and insert statistics for whole-genome sequencing.

Aligment Coverage

shCtrl						
**% Coverage**	%>1X	%>5X	%>10X	%>15X	%>20X	%>30X
**Value**	98.9	98.6	98.4	98.0	97.1	89.2

shNKX6.3[#1]						
**% Coverage**	%>1X	%>5X	%>10X	%>15X	%>20X	%>30X
**Value**	98.9	98.6	98.4	97.7	96	88.9

shNKX6.3[#2]						
**% Coverage**	%>1X	%>5X	%>10X	%>15X	%>20X	%>30X
**Value**	98.9	98.6	98.3	97.1	94.7	84.6

*% Coverage: The percentage of bases in non-N-reference regions with specific depth of coverage or greater

Insert Statistics

	shCtrl	shNKX6.3[#1]	shNKX6.3[#2]
**Fragment length median**	460 bp	462 bp	469 bp
**Standard deviation**	110.4 bp	112.0 bp	114.1 bp

(**C**) Summary of single nucleotide variants.

SNP & INDEL

	shCtrl			shNKX6.3[#1]			shNKX6.3[#2]		
	SNPs	Small insertions	Small deletions	SNPs	Small insertions	Small deletions	SNPs	Small insertions	Small deletions
**# of variants**	3,544,930	286,115	282,482	3,501,785	286,944	280,713	3,492,917	280,173	277,100
**# of synonymous vaiants**	11,122	-	-	11,193	-	-	11,170	-	-
**# of non-synonymous variants**	10,144	-	-	10,538	-	-	10,542	-	-
**# of splicing variants**	211			280			272		
**# of stop gained**	81			101			104		
**# of stop loss**	31			54			44		
**# of frame shift**	211			410			412		
**% found in dbSNP138**	95			94.1			94.1		
**% found in dbSNP142**	95.7			94.8			94.8		
**Het/Hom ratio**	1.34			1.26			1.27		
**Ts/Tv ratio**	2.011			2.0119			2.0107		

*Het/Hom ratio: Ratio of Number of heterozygous variants to Number of homozygous variants

*Ts/Tv ratio: Ratio of Transition rate of SNVs that pass the quality filters divided by transversion rate of SNVs that pass the quality filters. Transition rate of SNVs that pass the Quality filters divided by transversion rate of SNVs that pass the quality filters. Transitions are interchanges of purines (A, G) or of pyrimidines (C, T). Transversions are interchanges between purine and pyrimidine bases.

Supplementary Table S3. Gene set enrichment of NKX6.3 depletion-induced mutant genes.

15 biological processes were enriched by 721 genes with mutation on coding regions

Biological process	Fold enrichment	P-value (Hypergeometric test)	
Cell growth and/or maintenance	1.633013104	7.58456E-05	AGRN; PLCH2; MEGF6; CROCC; COL11A1; TNFAIP6; COL5A2; MAP2; COL4A4; DNAH12; FLNB; POPDC2; GOLGB1; CLDN16; CCNI; FRAS1; DNAH5; VCAN; PCDHA8; PCDHB8; DIAPH1; THG1L; COL19A1; COL28A1; ELMO1; LAMB1; PCM1; KAT6A; KIF24; DNAI1; COL27A1; KRTAP5-5; DCDC1; MYO7A; AMOTL1; TECTA; KRT74; KRT2; ARPC3; DNAH10; SPTBN5; DMXL2; MYO5A; KIF7; TMEM8A; MYO1C; MYO15A; KRT10; KRT38; TIMP2; TUBB4A; SPTBN4; KRTAP15-1; KRTAP10-6; KRTAP10-10; KRTAP10-11; MYH9; DSPP; FHDC1; PALLD; COL11A2; SCUBE2; MEGF11; NEFH;
DNA repair	0.994469384	0.004848889	MUTYH; NHEJ1;
Immune response	0.897391424	0.007157765	BCL9; GPA33; CR1; IL7R; HLA-C; HLA-B; HLA-DRB1; DSE; OAS1; IL4R; C3; FCGBP; PSG8; GP6; MAGEB1; MAGEC1; HLA-DQB2; SAGE1;
Apoptosis	0.523118618	0.009507347	CSRNP3; CASP8; TP53; CARD10; TAF4B
Cell cycle	2.62104357	0.017763121	CDC14B; NEK5;
Organogenesis	3.217324071	0.027329386	MUC6;
Signal transduction	1.028756704	0.036593405	UTS2; ARHGEF19; MACF1; PIK3R3; LPAR3; GBP1; VCAM1; PEX11B; FLG; FLG2; NPR1; CD1C; OR10J3; RGS2; NFASC; PLXNA2; USH2A; DISP1; OR2T33; OR2T10; OR2T27; DNAJC27; PLEK; MAP3K19; ARHGAP15; FAP; LRP2; IGFBP2; ITPR1; DLEC1; KIF15; SACM1L; CELSR3; RHOA; MST1; LRIG1; OR5H6; PIK3CA; AHSG; MUC20; MUC4; PAK2; WFS1; CLNK; SLIT2; TEC; CDKL2; CTNND2; PTGER4; ITGA1; RHOBTB3; MCC; APBB3; PCDH12; FAT2; OR14J1; MAP3K4; ADAP1; ELMO1; PKD1L1; TG; RECK; DCAF10; APBA1; ITGA8; AGAP4; SAR1A; TECTB; GRK5; MKI67; CDHR5; TSPAN32; OR52K2; OR51A2; SMPD1; SWAP70; OR4C3; OR4A16; BEST1; SORL1; OR8D2; PEX5; PIK3C2G; NELL2; ITGB7; LACRT; LRP1; LGR5; IFT88; NEK3; OR4L1; BMP4; NRXN3; BUB1B; ITGA11; CHRNA5; CCNF; TNFRSF12A; KDM8; IL4R; CDH1; PDPR; PLD2; PITPNM3; EFNB3; NF1; NBR1; OR4D2; SOCS3; CABYR; MATK; FFAR1; NINL; SNX21; DOK5; HRH3; TPTE; MICALL1; PPEF1; AR; MCF2; NOTCH2; SELP; CCR5; PTPN13; IBSP; GRID2; PDE5A; PLK4; NR3C2; DCHS2; GUCY1A3; ANKRD37; TLR3; FAT1; OR5M8; MS4A1; OR10G9; HCAR3; APOBR; CGB7;
Calcium-mediated signaling	6.402793448	0.03709086	CAMK1D; CABYR;

Cell adhesion	2.662329314	0.039381166	USH2A; FAT3; PCDH20; FHDC1; SCARF2; MADCAM1
Cell proliferation	1.478830273	0.039619471	LACRT; IL4R; WT1
Cell migration	1.931251824	0.041265312	ELMO1; FHDC1
Cell differentiation	0.999244739	0.042701981	WT1;
Cellular morphogenesis	14.42193526	0.048470613	CYLC1;
Intercellular junction assembly and/or maintenance	14.42193526	0.048470613	PKP1;
Vitamin/cofactor transport	14.42193526	0.048470613	SLC46A1;

Supplementary Table S4. APOBEC and AICDA mutation patterns in HFE-145$^{shNKX6.3}$ cells.

Sample	shNKX6.3$^{\#1}$	shNKX6.3$^{\#2}$
mutations	4186	3083
insertions	702	776
deletions	600	634
indels	1302	1410
frame shift	95	104
substitutions	2885	3013
A_to_T	105	107
A_to_G	374	407
A_to_C	167	169
A	646	683
a	13882	14348
T_to_A	99	91
T_to_C	375	407
T_to_G	179	180
T	653	678
t	14271	14830
G_to_C	106	112
G_to_T	152	151
G_to_A	516	556
G	774	819
g	14688	15565
C_to_G	129	135
C_to_A	127	143
C_to_T	555	555
C	811	833
c	14839	15517
tCw_to_G	19	21
tCw_to_A	19	21
tCw_to_T	57	65
tCw	95	107
tcw	1637	1740
tCw_per_mut	0.032928943	0.011284434
tCw_per_C	0.041923551	0.040816327
tcw_per_c	0.110317407	0.112135078

**enrich_tCw**	1.061839083	1.145505789
**freq_tCw**	0.058032987	0.061494253
**wGa_to_C**	20	16
**wGa_to_T**	21	19
**wGa_to_A**	51	47
**wGa**	92	82
**wga**	1634	1717
**wGa_per_mut**	0.031889081	0.0272154
**wGa_per_G**	0.118863049	0.1001221
**wga_per_g**	0.111247277	0.110311597
**enrich_wGa**	1.068458057	0.907629871
**freq_wGa**	0.05630355	0.047757717
**[tCw_to_G+tCw_to_T]_per_mut**	0.050953206	0.049452373
**tCw_to_G+tCw_to_T**	147	149
**[(C_to_G)+(C_to_T)]-[(tCw_to_G)+(tCw_to_T)]**	1159	1209
**tcw+wga**	3271	3457
**c-tcw**	26256	27625
**APOBEC_enrich**	1.065003708	1.028635096
**tCw_to_G_enrich**	0.222113073	0.201373008
**tCw_to_A_enrich**	0.22780828	0.217700549
**tCw_to_T_enrich**	0.615082356	0.609561538
**wrC_to_G**	27	31
**wrC_to_A**	27	35
**wrC_to_T**	120	121
**wrC**	174	187
**wrc**	2536	2659
**wrC_per_mut**	0.060311958	0.011284434
**wrC_per_C**	0.041923551	0.040816327
**wrc_per_c**	0.170901004	0.171360443
**enrich_wrC**	1.255404785	1.310044439
**freq_wrC**	0.068611987	0.070327191
**Gyw_to_C**	17	26
**Gyw_to_T**	48	41
**Gyw_to_A**	123	137
**Gyw**	188	204
**gyw**	2641	2801
**Gyw_per_mut**	0.065164645	0.067706605
**Gyw_per_G**	0.242894057	0.249084249

**gyw_per_g**	0.179806645	0.179955027
**enrich_Gyw**	1.350862517	1.384147211
**freq_Gyw**	0.071185157	0.072831132
**[wrC_to_G+wrC_to_T]_per_mut**	0.099480069	0.050448058
**wrC_to_G+wrC_to_T**	287	152
**[(C_to_G)+(C_to_T)]-[(wrC_to_G)+(wrC_to_T)]**	1019	1043
**wrc+gyw**	5177	5460
**c-wrc**	24350	25622
**AICDA_enrich**	1.302628162	1.34735807
**wrC_to_G_enrich**	0.158330495	0.196417928
**wrC_to_A_enrich**	0.269881525	0.261890571
**wrC_to_T_enrich**	0.874416142	0.88904957

Supplementary Table S5. Gene set enrichment of NKX6.3 depletion-induced mutant genes.

(A) Gene set enrichment of APOBEC-induced mutant genes.

**Biological process**	**Fold enrichment**	**P-value (Hypergeometric test)**	
Cell migration	17.02352368	0.027690502	ELMO1;
Cell cycle	11.60940892	0.034763857	CDC14B;
Regulation of cell proliferation	11.10487138	0.037102781	SHOX;
RNA metabolism	9.824032694	0.039789722	WAC;
Lipid metabolism	7.740778261	0.042259684	PON2;
Apoptosis	3.46124907	0.026535735	TP53; SHOX; NLRP2;
Cell growth and/or maintenance	1.57641447	0.045720555	DNAH12; LPIN2; KRTAP10-11; THG1L; ELMO1; COL1A2; FAT4;
Immune response	1.322041992	0.039894458	MAGEB1; HLA-B; MAGEB2;
Transport	1.043197772	0.029128956	CPNE5; TTYH3; SCN8A; SLC5A12; VAMP3;
Signal transduction	0.90097233	0.013720484	STK40; MCC; RRAGD; FAM3C; ADRB1; SMAD2; RSPO4; SELP; PIK3CA; SEMA5A; ELMO1; CHRNA5; WNT3; MN1;

(B) Gene set enrichment of AICDA-induced mutant genes.

**Biological process**	**Fold enrichment**	**P-value (Hypergeometric test)**	
Cellular morphogenesis	65.72913983	0.015230571	CYLC1;
Transmembrane receptor protein tyrosine kinase signaling pathway	65.72913983	0.015230571	DDR2;
Vitamin/cofactor transport	65.72913983	0.015230571	SLC46A1;
Vitamin metabolism	65.72913983	0.015230571	RETSAT;
Muscle contraction	26.37037346	0.03764548	AHNAK;

Cell cycle	6.002524809	0.015541797	GAS7;
Regulation of nucleobase, nucleoside, nucleotide and nucleic acid metabolism	1.018056156	0.013559796	GLIS1; ZNF697; POGZ; IRF2BP2; ZNF595; CREBRF; ZNF460; HMGN1; ZBED1; CENPE; LARP1B; MAML3; ADARB2; ZNF705A; GCM1; PRIM2; RB1CC1; VAX1; ZNF676; POLA1; PHF8; LEF1;
Metabolism	0.933445451	0.042689621	CYP4A11; CYP26B1; DPAGT1; FTCD; PRODH; ETFDH; NADK; OGDH; GLYAT; FUT3; TTLL12; DSEL;
Cell growth and/or maintenance	0.931341094	0.034138731	KRT74; MYO5A; TMEM8A; EMD; COL11A1; MYOF; KIF7; COL25A1;
Signal transduction	0.864843499	0.025490486	FAM3C; AGAP4; FFAR1; YWHAB; FANCD2; CCR5; MUC20; NR3C2; SORBS2; TLR3; IGF1R; ARHGEF19; BCL10; NOS1AP; OR2T33; NOTCH1; PLXDC2; SORL1; NRXN3; CEACAM6; PDLIM5; RRH; PDE5A; DCHS2; NPY2R; FAT1;

Supplementary Table S6. Primers used in this study.

Assay	Gene		Oligonucleotides
Real-time RT-PCR	NKX6.3	Forward	5'- TCTTTCTGCTTCTGGGGTGT -3'
		Reverse	5'- AGCGGCTTGTTGTACTCGTC -3'
	APOBEC3A	Forward	5'- ATGGCATTGGAAGGCATAAG -3'
		Reverse	5'- CAAAGAAGGAACCAGGTCCA -3'
	APOBEC3B	Forward	5'- TGTCACCCTGACCATCTCTG -3'
		Reverse	5'- GCATGAATTGCTGACCTTCA -3'
	APOBEC3C	Forward	5'- CAACGATCGGAACGAAACTT -3'
		Reverse	5'- TATGTCGTCGCAGAACCAAG -3'
	APOBEC3G	Forward	5'- ACCCTGACCATCTTTGTTGC -3'
		Reverse	5'- TTGGCTGTACACGAACTTGC -3'
	APOBEC3H	Forward	5'- GCTTCCCAGAGTTTGCTGAC -3'
		Reverse	5'- GCACGTACCCCTGGAATCTA -3'
	Ung	Forward	5'- AGGTTGTCATCCTGGGACAG -3'
		Reverse	5'- GCGTTGAGAAGGAGAACACC -3'
	Apex1	Forward	5'- GCTGCCTGGACTCTCTCATC -3'
		Reverse	5'- CGAGTCAAATTCAGCCACAA -3'
	AICDA	Forward	5'- CGCGCCTCTACTTCTGTGAG -3'
		Reverse	5'- TTCATCGTGTGTGACATTCCTG -3'
	NFkB p65	Forward	5'- AGTACCTGCCAGATACAGACGAT -3'
		Reverse	5'- GATGGTGCTCAGGGATGACGTA -3'
	CBFβ	Forward	5'- TTTGAAGGCTCCCATGATTC -3'
		Reverse	5'- ATCTTCAAATTCGCGTGTCC -3'
	β-actin	Forward	5'- GTTGCTATCCAGGCTGTG -3'
		Reverse	5'- TGATCTTGATCTTCATTGTG -3'
ChIP-qPCR	APOBEC3B	Forward	5'- AAGAGCCATGGAGAAGGATG -3'
		Reverse	5'- TTGTGCACATGTACCCTAAAACTT -3'
	APOBEC3C	Forward	5'- CCCACCGGTTATTTCTTGGT -3'
		Reverse	5'- TGCTGAGGCAAGAGACTGAA -3'
	APOBEC3G	Forward	5'- AAAAGACTGTGGAGACCAAGG -3'
		Reverse	5'- GGGGGAAAATTTGCATCTGT -3'
	APOBEC3H	Forward	5'- CACTGTCAGCTTGCTTGCAT -3'
		Reverse	5'- CTCAGCCTCCCAAAGTGTTG -3'
	Ung	Forward	5'- TGCCGTGGTTTGAATATCTG -3'
		Reverse	5'- ACTCCCACGGTAACCCATTA -3'
	Apex1	Forward	5'- CCAGCCGTGTTTGGTACTTT -3'
		Reverse	5'- AGCTGGTCTCGAACTCCTGA -3'

	NFkB p65	Forward	5’- TCTGGGCCAGGTGTGGTGGC -3'
		Reverse	5’- TGGCCTCACAAAGTGCTGGGA -3'
	CBFβ	Forward	5’- CTGAGACTACAGGCGTGTGC -3'
		Reverse	5’- GTCAGGCTGGTCTCGAACTC -3'
Luciferase activity	APOBEC3B	Forward	5’- GGTACCTGGGCAACAGAGTGAGACTCTGTC -3'
		Reverse	5’- CTCGAGACTTAAAGTATAATAATAATAAAAAAAAAGAAA -3'
	APOBEC3C	Forward	5’- GGTACCCACTTCTCACAACAGCCCTTCAGC -3'
		Reverse	5’- CTCGAGGAGGCAAGAGACTGAAGGCACAAA -3'
	APOBEC3G	Forward	5’- GGTACCCAAGTTAAGATAAAAGACTGTGGAGAC -3'
		Reverse	5’- CTCGAGTCTTTGCCATATCTGAAATGGCCCT -3'
	APOBEC3H	Forward	5’- GGTACCTTACAGGTGTGAGCCACCATGCC -3'
		Reverse	5’- CTCGAGCTGATCTGCCCACCTCAGCCTC -3'
	Ung	Forward	5’- GGTACCATAGTTTTGCCTTTTCCAGAATTTCATA -3'
		Reverse	5’- CTCGAGCATCACATCGCGAGGGGCCTGA -3'
	Apex1	Forward	5’- GGTACCAATTCTCCTGCCTCAGCCTCCCA -3'
		Reverse	5’- CTCGAGCCAGGCCAAGCTGGTCTCGAAC -3'
	NFkB p65	Forward	5’- GGTACCTCTGGGCCAGGTGTGGTGGC -3'
		Reverse	5’- CTCGAGTGGCCTCACAAAGTGCTGGGA -3'
	CBFβ	Forward	5’- GGTACCGTTCAAGCGATTCTCCTGCCTCAG -3'
		Reverse	5’- CTCGAGTGGAGTGCAATGGCGCGATCTCG -3'
Sequencing	PIK3CA	Forward	5’- TGGCCAGTACCTCATGGATT -3'
		Reverse	5’- TTGGCATGCTCTTCAATCAC -3'
	TP53	Forward	5’- AGCTGTTCCGTCCCAGTAGA -3'
		Reverse	5’- GTGGAAGGAAATTTGCGTGT -3'
	CDH1	Forward	5’- TGGACAGGGAGGATTTTGAG -3'
		Reverse	5’- ACCTGAGGCTTTGGATTCCT -3'
	RhoA	Forward	5’- TTCTGGGGTCCACTTTTCTG -3'
		Reverse	5’- AAGGACCAGTTCCCAGAGGT -3'
	EP300 (K350R)	Forward	5’- CTCCTTTTGCATGCTCACAA -3'
		Reverse	5’- TTTTTGAGGGGGAGACACAC -3'
	EP300 (E643stop)	Forward	5’- TGCATATGCTCGGAAAGTTG -3'
		Reverse	5’- CCCTGGATTCATGGAAACTG -3'
	EP300 (E1025stop)	Forward	5’- TTCCCAGGAAGTGAAGATGG -3'
		Reverse	5’- GGCTGGTCTTCCTCCTCTTT -3'
	Casp8 (S316P)	Forward	5’- GCATTAGGGACAGGAATGGA -3'
		Reverse	5’- GCCATAGATGATGCCCTTGT -3'
	Casp8 (S424T)	Forward	5’- GGATGAGGCTGACTTTCTGC -3'
		Reverse	5’- GCATCTGTTTCCCCATGTTT -3'

POLA1	Forward	5’- TCAAATGTCCATGCCCTACA -3'
	Reverse	5’- GAGTTCGATTGCGACAGGTT -3'
FRET-deaminase activity		5′-(6-FAM)-AAATTCTAATAGATAATGTGA-(TAMRA)-3′

Supplementary Table S7. List of antibodies used in this study.

Name	Company	Cat No.
Anti-NKX6.3 (IHC & Western blot & IF)	Atlas antibodies	HPA042790
Anti-APOBEC3A (Western blot)	Abcam	ab150369
Anti-APOBEC3B (Western blot & IF)	Abcam	ab191695
Anti-APOBEC3C (Western blot)	Abcam	ab209560
Anti-APOBEC3G (Western blot)	Abcam	ab109727
Anti-Ung (Western blot)	Abcam	ab62520
Anti-Apex1 (Western blot)	Abcam	ab92744
Anti-SMUG1 (Western blot)	Abcam	ab192240
Anti-Rev1 (Western blot)	Santa Cruz	sc-393022
Anti-XRCC1 (Western blot)	Miilipore	DR1103
Anti-LIG3 (Western blot)	Miilipore	MABE1011
Anti-NFκB p65 (Western blot & IF)	Santa Cruz	sc-109
Anti-p-NFκB p65 (Western blot)	Cell signaling	#3033
Anti-CBFβ (Western blot & IF)	Cell signaling	#62184
Anti-AICDA (Western blot & IF & IHC)	Cell signaling	#4975
Anti-E-cadherin (Western blot & IF)	BD Biosciences	610404
Anti-p53 (Western blot & IF & IHC)	Cell signaling	#2524
Anti-p21 (Western blot & IF)	Cell signaling	#2946
Anti-RhoA-GTP (Western blot)	NewEast Biosciences	26904
Anti-RhoA (Western blot & IF)	Abcam	ab54835
Anti-PIK3CA (Western blot & IF)	Cell signaling	#4249
Anti-p-Akt (Western blot & IF)	Cell signaling	#4058
Anti-Akt (Western blot)	Cell signaling	#2920
Anti-EP300 (Western blot & IF & IHC)	Santa Cruz	sc-585
Anti-Caspase 8 (Western blot)	Cell signaling	#9746
Anti-PARP (Western blot)	Cell signaling	#9532
Anti-Fas (apoptosis induction)	Miilipore	05-201
Anti-GAPDH (Western blot)	Santa Cruz	sc-32233

Supplementary Figures

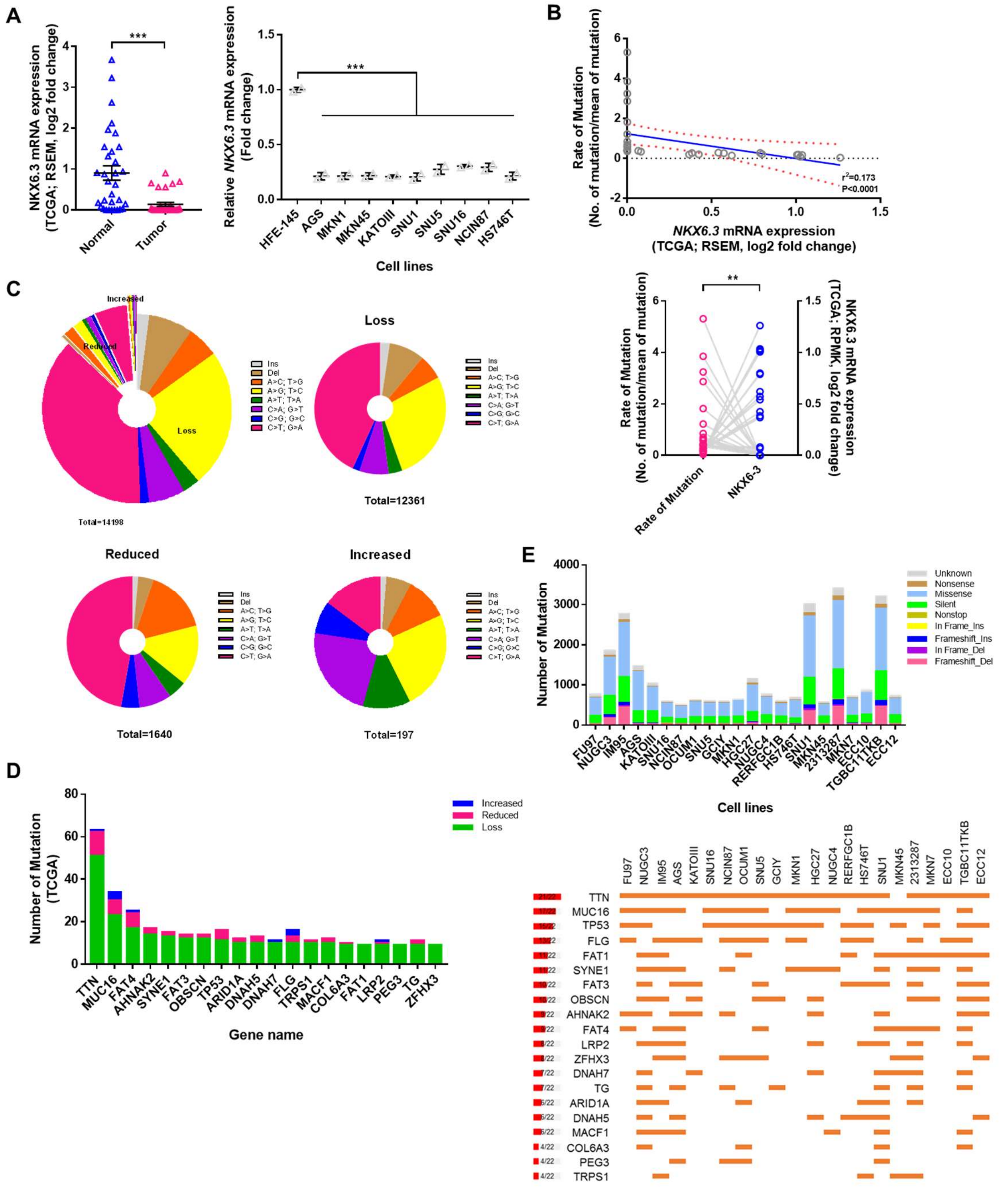

Supplementary Figure S1. NKX6.3 expression and genetic mutations in gastric cancers from TCGA and COSMIC data sets. (A) Compared with non-neoplastic gastric mucosae (n=32) and HFE-145 immortalized gastric epithelial cells, gastric cancer

tissues (n=32) and cancer cell lines (n=9) showed reduced NKX6.3 expression in TCGA and COSMIC data sets. **(B)** Inverse correlation between NKX6.3 expression and mutation rate in gastric cancer tissues (n=32) and cancer cell lines (n=9) (Spearman rank correlation test, $P < 0.0001$). Mean of mutation is a mean number of point mutations detected in 32 TCGA gastric cancer samples. **(C)** Mutation spectrum of eight insertion, deletion, transition and transversion categories for 32 TCGA gastric cancer tissues. A high frequency of G to A transition mutations was observed the gastric cancer tissues with loss (43.2%, $P < 0.0001$) or reduced (47.2%, $P < 0.0001$) or increased (14.7%, P=0.27) NKX6.3 expression. **(D)** The number of coding region mutations in 20 representative genes with point mutations in the gastric cancer tissues from TCGA data sets. **(E)** The COSMIC data from gastric cancer cell lines (n=22) showed type of mutations (upper), and coding region point mutations in 20 representative genes (lower) in each cell lines. *** P<0.001, ** P<0.01, based on the student`s *t*-test and Pearson correlation test.

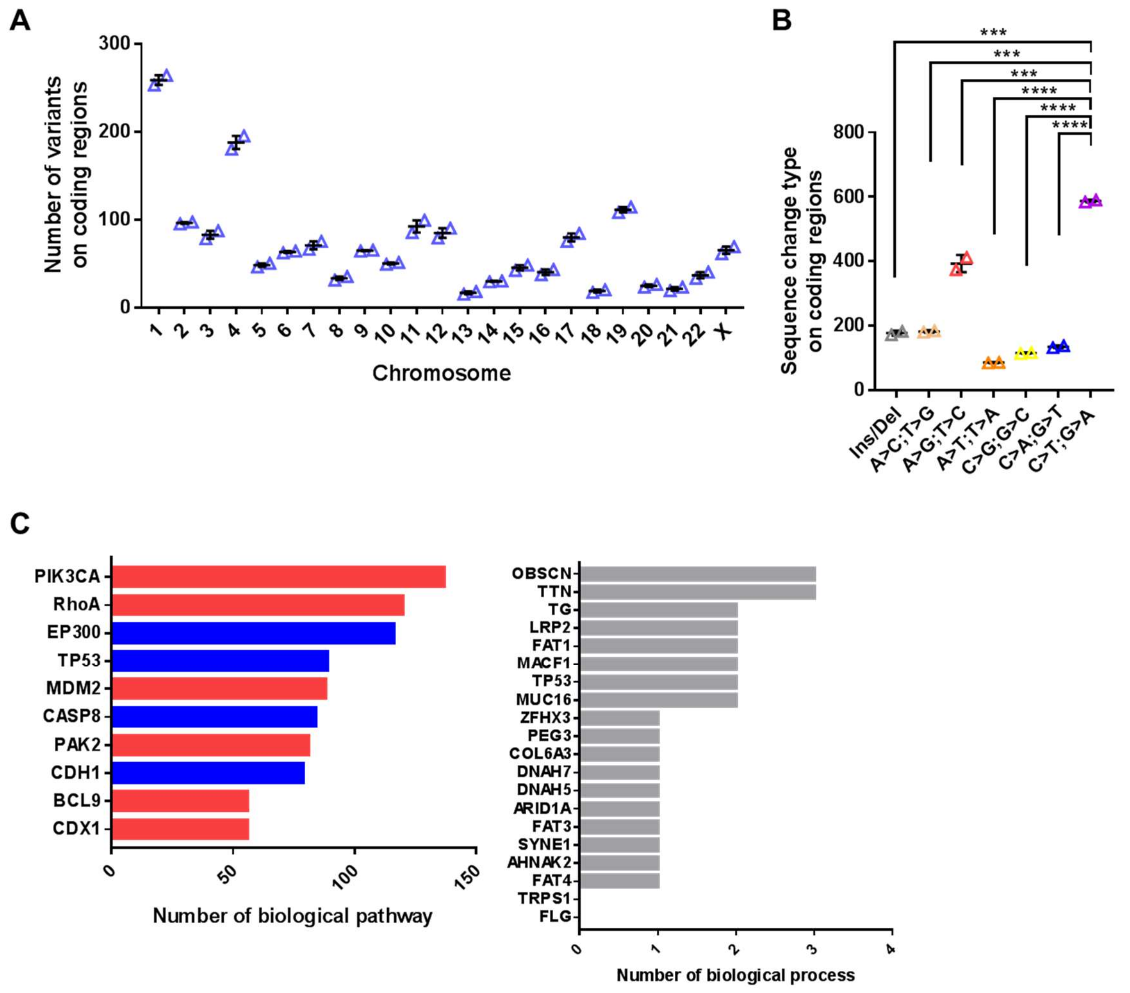

Supplementary Figure S2. Depletion of NKX6.3 enhances mutations in the coding regions. (A) Number of variants in the gene coding regions at each chromosome in HFE-$145^{shNKX6.3\#1}$ and HFE-$145^{shNKX6.3\#2}$ cells, compared to HFE-145^{shCtrl} cells. **(B)** Type of nucleotide changes observed in HFE-$145^{shNKX6.3\#1}$ and HFE-$145^{shNKX6.3\#2}$ cells. Depletion of NKX6.3 significantly enhanced frequency of C to T or G to A transition. **(C)** Number of biological pathway of 10 representative genes with coding region point mutations in HFE-$145^{shNKX6.3\#1}$ and HFE-$145^{shNKX6.3\#2}$ cells, and 20 genes with coding region point mutations in the 22 COSMIC gastric cancer cell lines.

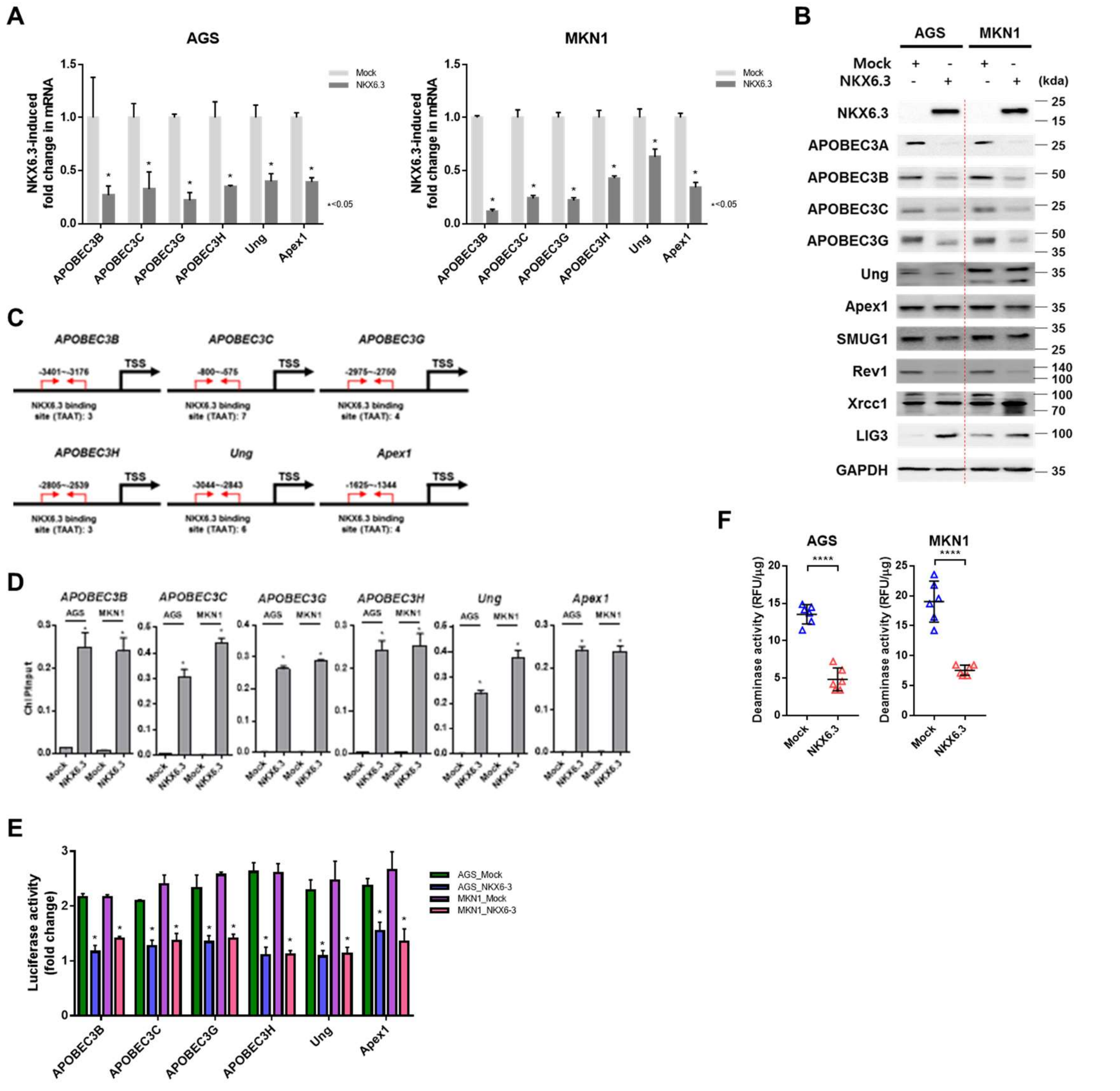

Supplementary Figure S3. NKX6.3 regulates APOBEC gene family expression. (A) NKX6.3 expression reduced mRNA expression of *APOBEC3B*, *3C*, *3G*, *3H*, *Ung* and *Apex1* in AGS$^{NKX6.3}$ and MKN1$^{NKX6.3}$ cells. **(B)** NKX6.3 regulated protein expression of APOBEC3A, 3B, 3C, 3G, Ung, Apex1, SMUG1, Rev1, Xrcc1 and LIG3 in AGS$^{NKX6.3}$ and MKN1$^{NKX6.3}$ cells. **(C)** Putative NKX6.3 binding motifs in the promoter regions from -0.5 kb to -3.5 kb relative to the transcription start site of *APOBEC3B, 3C, 3G, 3H, Ung* and *Apex1* genes. **(D)** NKX6.3 binding activity to *APOBEC3B, 3C, 3G, 3H, Ung* and *Apex1* genes in AGS$^{NKX6.3}$ and MKN1$^{NKX6.3}$ cells by ChIP-qPCR. **(E)** In luciferase

activity assay, mRNA transcript expression of those genes was significantly decreased in $AGS^{NKX6.3}$ and $MKN1^{NKX6.3}$ cells. **(F)** NKX6.3 inhibited deaminase activity in $AGS^{NKX6.3}$ and $MKN1^{NKX6.3}$ cells. Error bars represent ± SEM in three independent experiments. t-test, $*p < 0.05$.

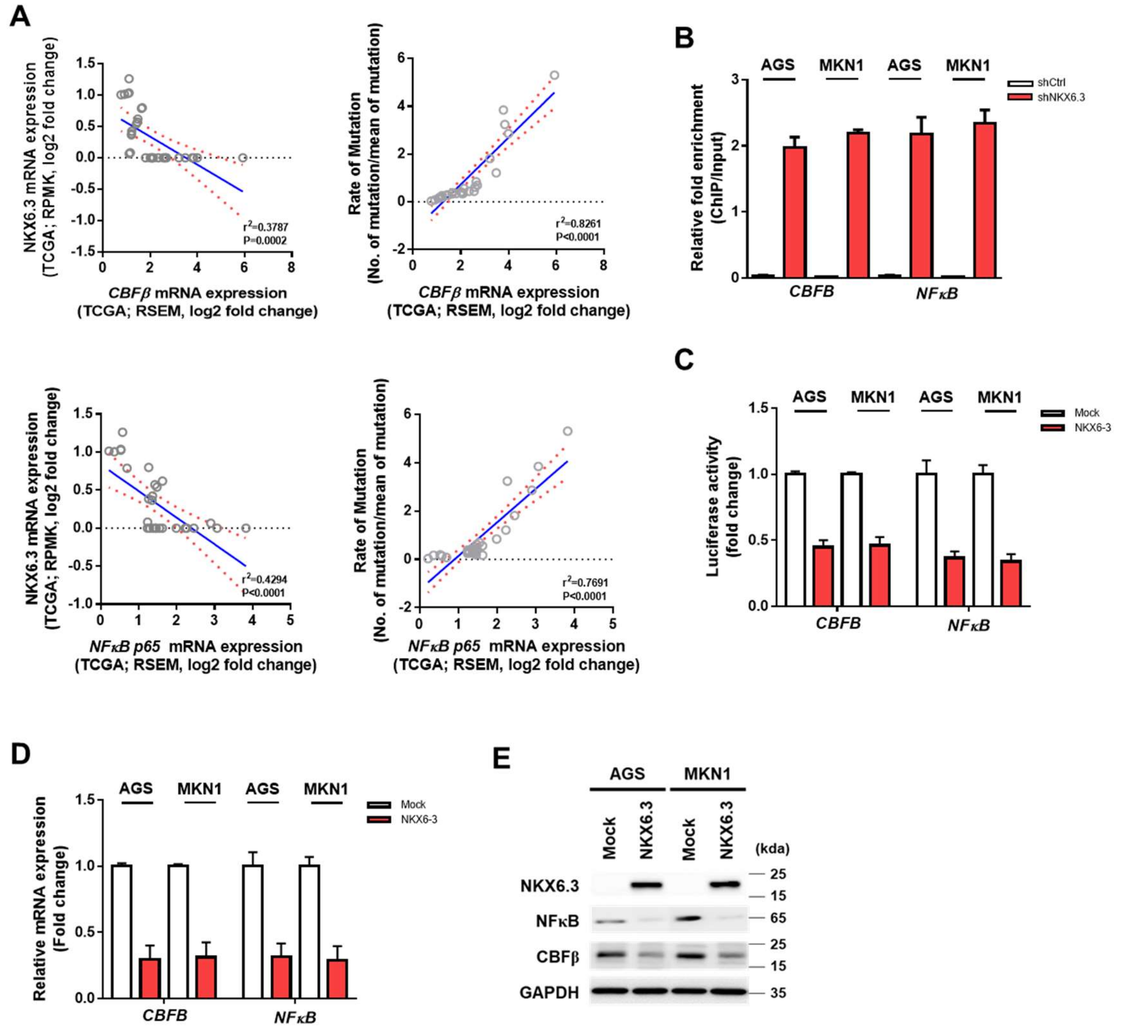

Supplementary Figure S4. NKX6.3 regulates NFκB and CBFβ expression. (A) The expression levels of *CBFβ* and *NFκB* were inversely associated with levels of NKX6.3 expression but positively associated with mutation rates in TCGA datasets **(B)** In a ChIP assay, the NKX6.3 binding activity to *NFκB* and *CBFβ* genes was found in $AGS^{NKX6.3}$ and $MKN1^{NKX6.3}$ cells. **(C)** In the luciferase activity assay, mRNA transcript expression of *CBFβ* and *NFκB* were significantly increased in $AGS^{NKX6.3}$ and $MKN1^{NKX6.3}$ cells. **(D, E)** In real-time RT-PCR and western blot analyses, mRNA transcript (D) and protein (E) expression of *CBFβ* and *NFκB* were significantly increased in $AGS^{NKX6.3}$ and $MKN1^{NKX6.3}$ cells.

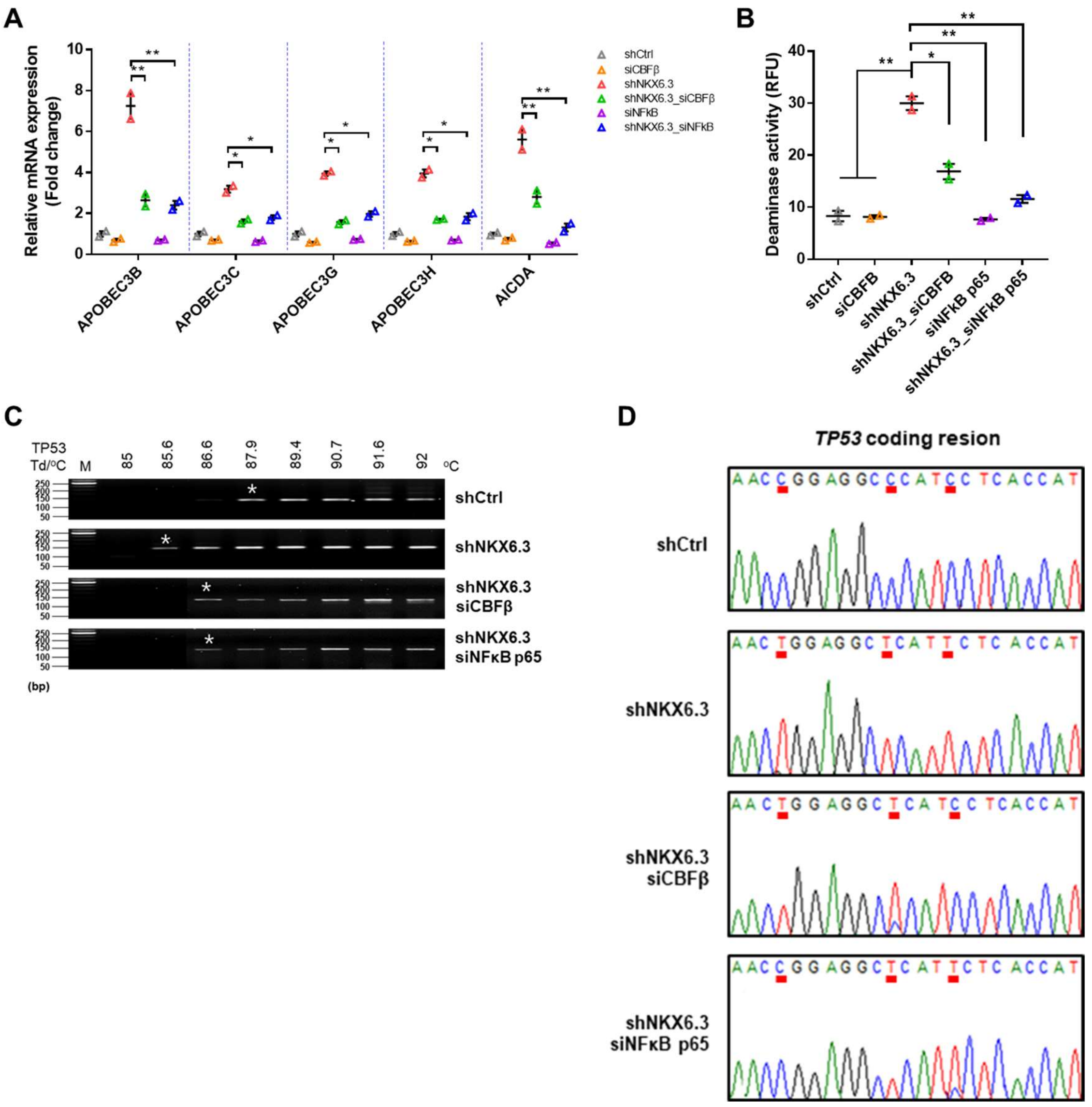

Supplementary Figure S5. Effects of CBFβ and NFκB p65 on NKX6.3 depletion-induced mutations. (A) Knockdown of *CBFβ* or *NFκB p65* significantly decreased mRNA expression of the AICDA/APOBEC gene family NKX6.3 depleted HFE-145 cells. **(B)** Knockdown of *CBFβ* or *NFκB p65* significantly inhibited deaminase activity in NKX6.3 depleted HFE-145 cells. **(C)** 3D-PCR products were recovered as low as 83.4ºC for the HFE-145$^{shNKX6.3}$ cells, compared to 87.9ºC for the HFE-145^{shCtrl} cells. In HFE-145$^{shNKX6.3}$ cells with si*CBFβ* or si*NFκB p65* were partially recovered, compared to HFE-

145$^{shNKX6.3}$ cells. **(D)** Sequence in *TP53* coding region.

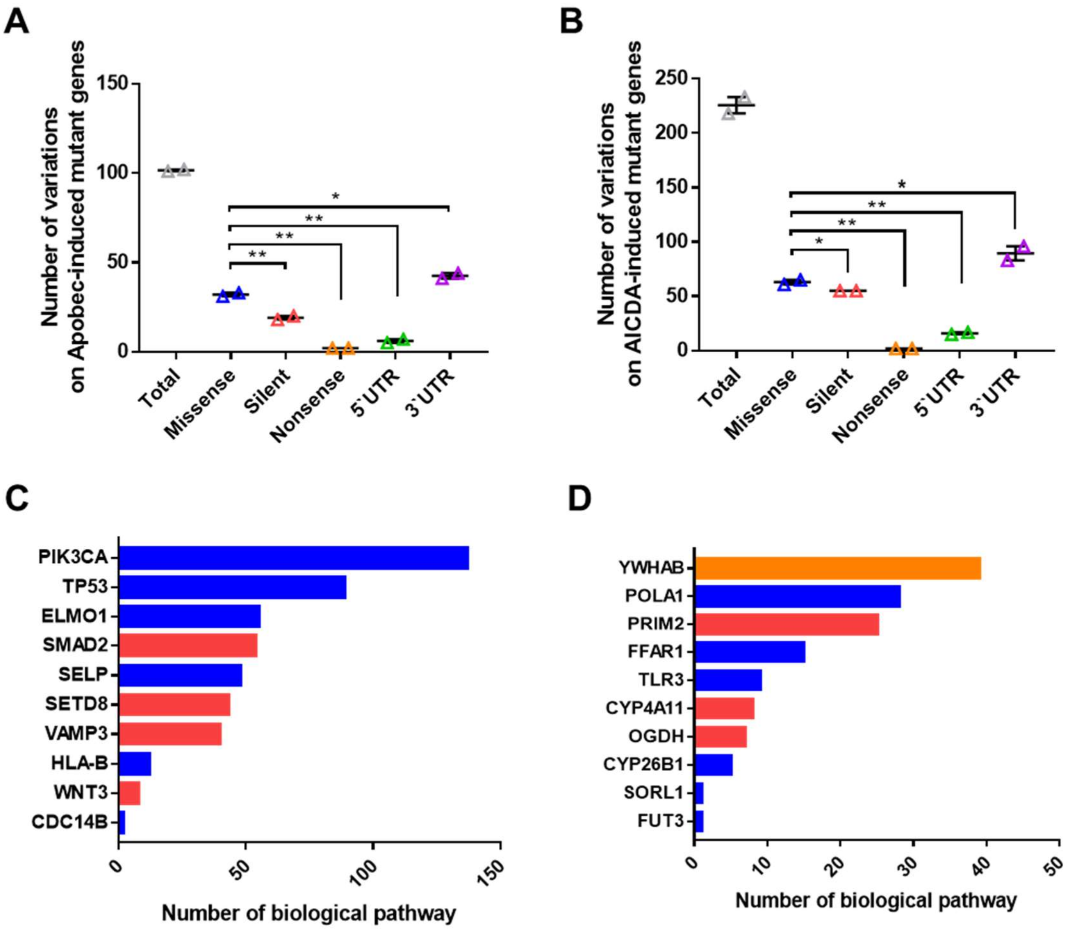

Supplementary Figure S6. NKX6.3 depletion induces the AICDA/APOBEC mutagenesis pattern. (A, B) In HFE-145$^{shNKX6.3}$ cells, the genes with APOBEC (A) and AICDA (B) mutation patterns frequently had missense mutations in gene coding region and mutations in the 3`-UTR. **(C)** Frequently APOBEC-induced mutant genes. **(D)** Frequently AICDA-induced mutant genes.

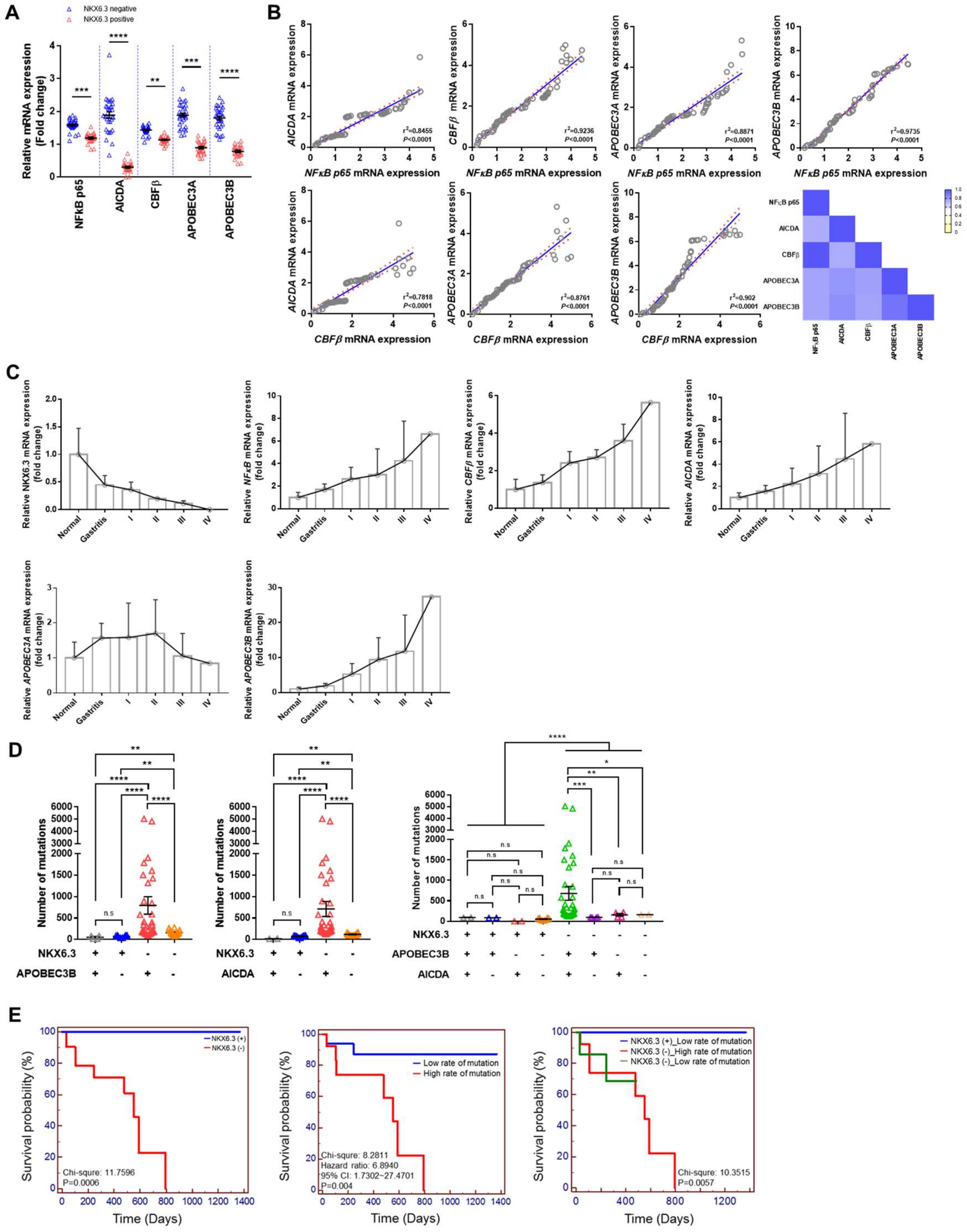

Supplementary Figure S7. Correlation with *AICDA/APOBEC gene family*, *NFκB p65* and *CBFβ* in both gastric cancers and non-cancerous gastric mucosae. (A) NKX6.3 expression was inversely correlated with expression of *AICDA*, *NFκB p65*,

CBFβ, *APOBEC3A* and *APOBEC3B* in 55 non-cancerous gastric mucosae tissues samples. **(B)** Expression of *AICDA*, *APOBEC3A* and *APOBEC3B* was positively correlated with *NFκB p65* and *CBFβ.* **(C)** Depletion of NKX6.3 and increased expression of *NFκB p65*, *CBFβ*, *AICDA*, *APOBEC3A* and *APOBEC3B* genes at mRNA of levels of in gastric mucosae with gastritis, compared with normal gastric mucosae, and gastric cancers with higher TNM stage, compared those with lower TNM stage. **(D)** In 32 gastric cancers from the TCGA datasets, cancer tissues without NKX6.3 and with APOBEC3B and/or AICDA expression showed significantly higher mutations than those of the cases with NKX6.3 expression. **(E)** Kaplan-Meier analysis revealed that gastric cancer patients with negative NKX6.3 expression and/or high levels of mutation rate had shorter overall survival time, when compared to that of patients with positive NKX6.3 expression and/or low levels of mutation rates.

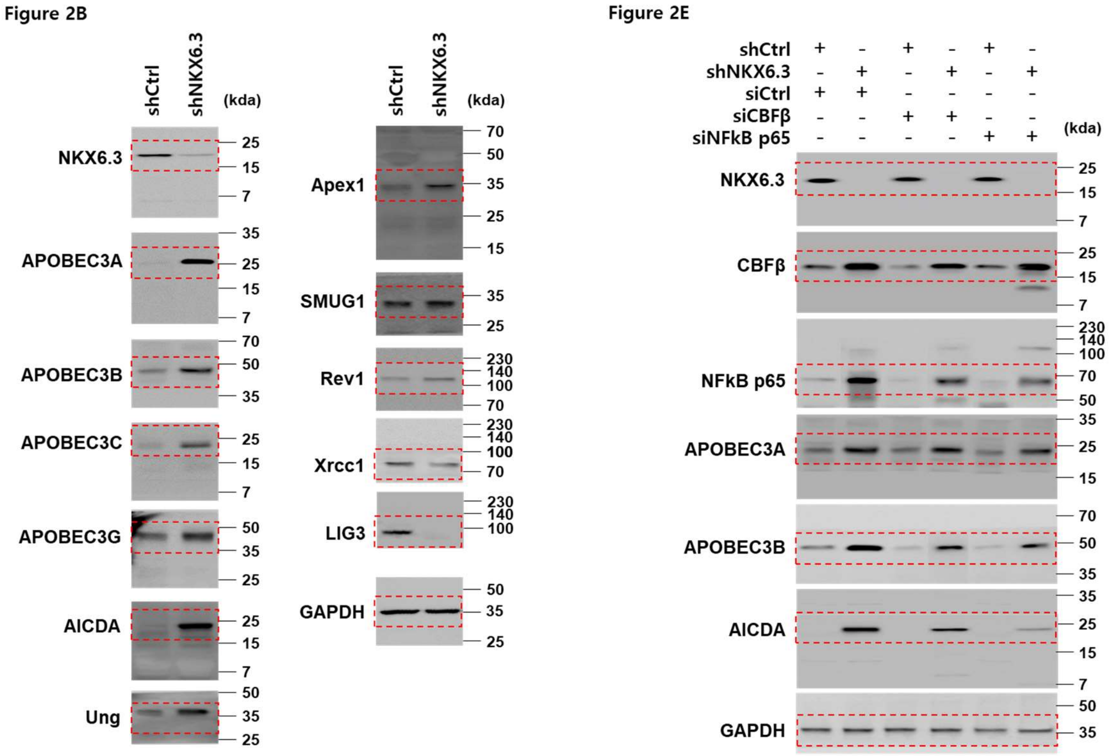

Supplementary Figure S8. Uncropped gel image for Figure 2.

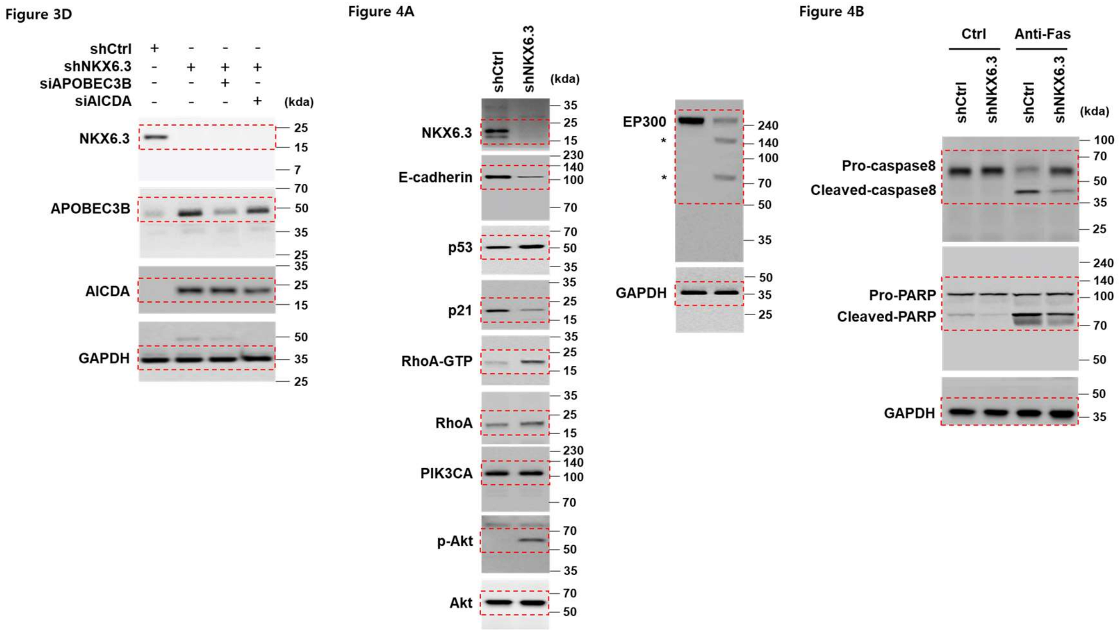

Supplementary Figure S9. Uncropped gel image for Figure 3 and 4.

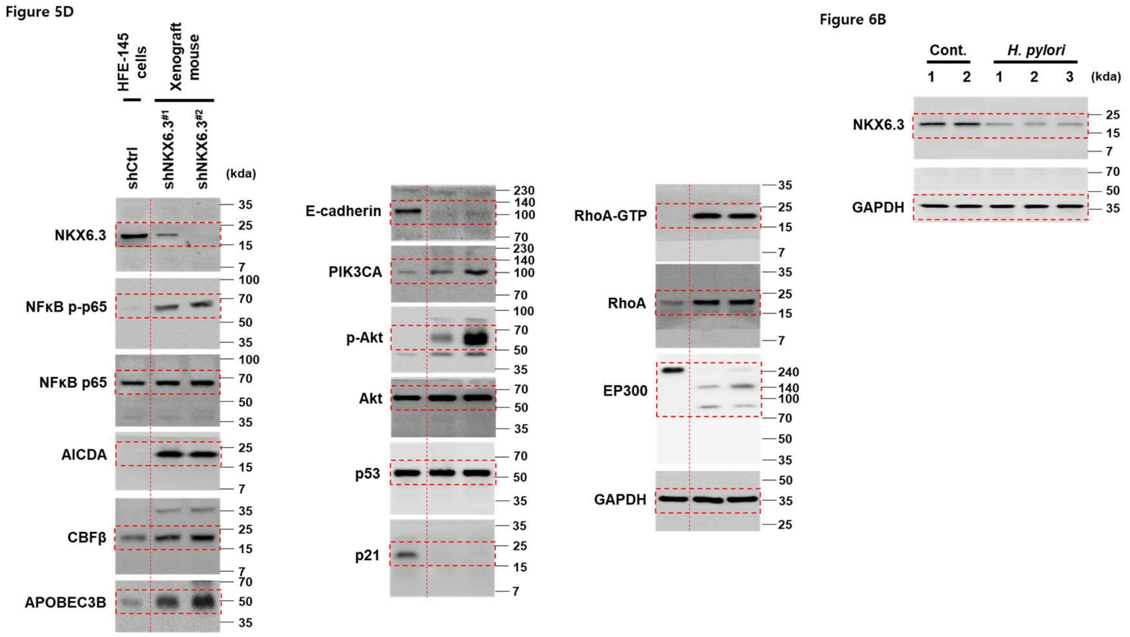

Supplementary Figure S10. Uncropped gel image for Figure 5 and 6.

Supplementary Figure S3B

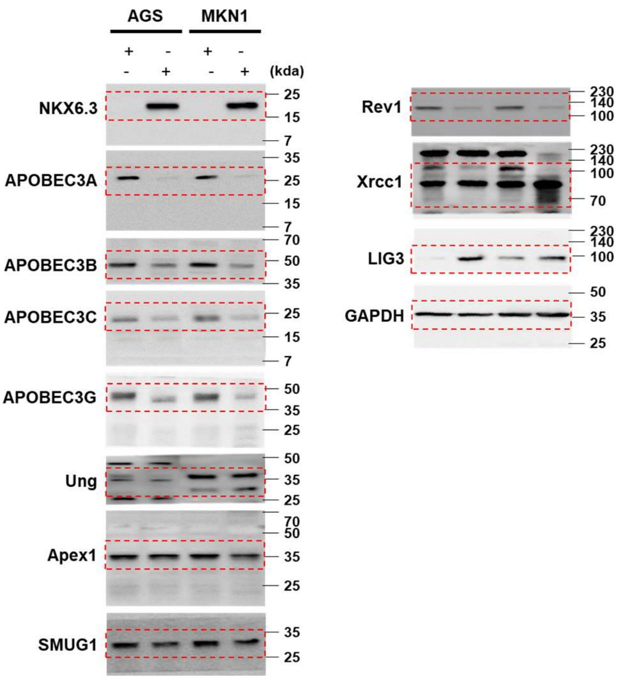

Supplementary Figure S4E

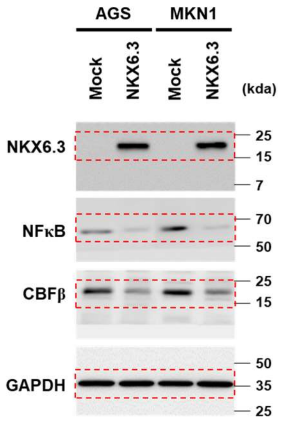

Supplementary Figure S11. Uncropped gel image for Supplementary Figure S3 and S4.

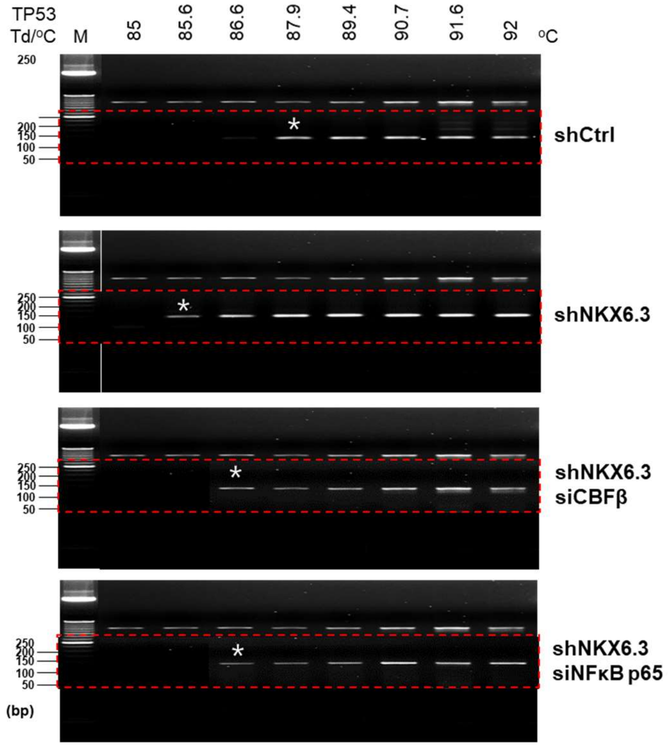

Supplementary Figure S12. Uncropped gel image for Supplementary Figure S5.
